# Flufenamic acid inhibits pyroptosis in ischemic flaps via the AMPK-TRPML1-Calcineurin signaling pathway

**DOI:** 10.1093/burnst/tkaf007

**Published:** 2025-02-17

**Authors:** Liang Chen, Ningning Yang, Kongbin Chen, Yingying Huang, Xian Liu, Gaoxiang Yu, Fulin Wang, Yong Gou, Yi Wang, Xiaolang Lu, Yuqi Wang, Lipeng Zhu, Weiyang Gao, Jian Ding

**Affiliations:** Department of Orthopaedics, The Second Affiliated Hospital and Yuying Children’s Hospital of Wenzhou Medical University, No. 109 WestXueyuan Road, Lucheng District, Wenzhou 325027, China; Zhejiang Provincial Key Laboratory of Orthopaedics, No. 109 WestXueyuan Road, Lucheng District, Wenzhou 325027, China; The Second Clinical Medical College of Wenzhou Medical University, No. 109 WestXueyuan Road, Lucheng District, Wenzhou 325027, China; Department of Orthopaedics, The Second Affiliated Hospital and Yuying Children’s Hospital of Wenzhou Medical University, No. 109 WestXueyuan Road, Lucheng District, Wenzhou 325027, China; Zhejiang Provincial Key Laboratory of Orthopaedics, No. 109 WestXueyuan Road, Lucheng District, Wenzhou 325027, China; The Second Clinical Medical College of Wenzhou Medical University, No. 109 WestXueyuan Road, Lucheng District, Wenzhou 325027, China; Department of Orthopaedics, The Second Affiliated Hospital and Yuying Children’s Hospital of Wenzhou Medical University, No. 109 WestXueyuan Road, Lucheng District, Wenzhou 325027, China; Zhejiang Provincial Key Laboratory of Orthopaedics, No. 109 WestXueyuan Road, Lucheng District, Wenzhou 325027, China; The Second Clinical Medical College of Wenzhou Medical University, No. 109 WestXueyuan Road, Lucheng District, Wenzhou 325027, China; Department of Orthopaedics, The Second Affiliated Hospital and Yuying Children’s Hospital of Wenzhou Medical University, No. 109 WestXueyuan Road, Lucheng District, Wenzhou 325027, China; Zhejiang Provincial Key Laboratory of Orthopaedics, No. 109 WestXueyuan Road, Lucheng District, Wenzhou 325027, China; The Second Clinical Medical College of Wenzhou Medical University, No. 109 WestXueyuan Road, Lucheng District, Wenzhou 325027, China; Department of Orthopaedics, The Second Affiliated Hospital and Yuying Children’s Hospital of Wenzhou Medical University, No. 109 WestXueyuan Road, Lucheng District, Wenzhou 325027, China; Zhejiang Provincial Key Laboratory of Orthopaedics, No. 109 WestXueyuan Road, Lucheng District, Wenzhou 325027, China; The Second Clinical Medical College of Wenzhou Medical University, No. 109 WestXueyuan Road, Lucheng District, Wenzhou 325027, China; Department of Hand Surgery, Ningbo Sixth Hospital, No. 1059 Zhongshan East Road, Jiangdong District, Ningbo 315042, China; Department of Orthopaedics, The Second Affiliated Hospital and Yuying Children’s Hospital of Wenzhou Medical University, No. 109 WestXueyuan Road, Lucheng District, Wenzhou 325027, China; Zhejiang Provincial Key Laboratory of Orthopaedics, No. 109 WestXueyuan Road, Lucheng District, Wenzhou 325027, China; The Second Clinical Medical College of Wenzhou Medical University, No. 109 WestXueyuan Road, Lucheng District, Wenzhou 325027, China; Department of Orthopaedics, The Second Affiliated Hospital and Yuying Children’s Hospital of Wenzhou Medical University, No. 109 WestXueyuan Road, Lucheng District, Wenzhou 325027, China; Zhejiang Provincial Key Laboratory of Orthopaedics, No. 109 WestXueyuan Road, Lucheng District, Wenzhou 325027, China; The Second Clinical Medical College of Wenzhou Medical University, No. 109 WestXueyuan Road, Lucheng District, Wenzhou 325027, China; Department of Orthopaedics, The Second Affiliated Hospital and Yuying Children’s Hospital of Wenzhou Medical University, No. 109 WestXueyuan Road, Lucheng District, Wenzhou 325027, China; Zhejiang Provincial Key Laboratory of Orthopaedics, No. 109 WestXueyuan Road, Lucheng District, Wenzhou 325027, China; The Second Clinical Medical College of Wenzhou Medical University, No. 109 WestXueyuan Road, Lucheng District, Wenzhou 325027, China; Department of Orthopaedics, The Second Affiliated Hospital and Yuying Children’s Hospital of Wenzhou Medical University, No. 109 WestXueyuan Road, Lucheng District, Wenzhou 325027, China; School of nursing, Wenzhou medical university, No. 109 WestXueyuan Road, Lucheng District, Wenzhou 325027, China; The Fifth Affiliated Hospital of Zhengzhou University, No. 3 Kangfu Front Street, Zhengzhou 450015, China; Department of Orthopaedics, The Second Affiliated Hospital and Yuying Children’s Hospital of Wenzhou Medical University, No. 109 WestXueyuan Road, Lucheng District, Wenzhou 325027, China; Zhejiang Provincial Key Laboratory of Orthopaedics, No. 109 WestXueyuan Road, Lucheng District, Wenzhou 325027, China; The Second Clinical Medical College of Wenzhou Medical University, No. 109 WestXueyuan Road, Lucheng District, Wenzhou 325027, China; Department of Orthopaedics, The Second Affiliated Hospital and Yuying Children’s Hospital of Wenzhou Medical University, No. 109 WestXueyuan Road, Lucheng District, Wenzhou 325027, China; Zhejiang Provincial Key Laboratory of Orthopaedics, No. 109 WestXueyuan Road, Lucheng District, Wenzhou 325027, China; The Second Clinical Medical College of Wenzhou Medical University, No. 109 WestXueyuan Road, Lucheng District, Wenzhou 325027, China

**Keywords:** FFA, TFE3, Autophagy, Oxidative stress, Ischemic flaps

## Abstract

**Background:**

Ischemic injury is a primary cause of distal flap necrosis. Previous studies have shown that Flufenamic acid (FFA) can reduce inflammation, decrease oxidative stress (OS), and promote angiogenesis, suggesting its potential role in protecting flaps from ischemic damage. This study investigated the effects and mechanisms of FFA in enhancing the survival of ischemic flaps.

**Methods:**

The viability of ischemic flaps was evaluated using laser doppler blood flow (LDBF) and survival rates. We examined levels of pyroptosis, OS, transcription factor E3 (TFE3)-induced autophagy, and elements of the AMPK-TRPML1-Calcineurin pathway through western blotting (WB), immunofluorescence, molecular docking, cellular thermal shift assay (CETSA) and surface plasmon resonance.

**Results:**

The findings suggest that FFA significantly enhances the viability of ischemic flaps. The improvement in flap survival associated with FFA can be attributed to increased autophagy, diminished OS, and the suppression of pyroptosis. Notably, the promotion of autophagy flux and an augmented resistance to OS are instrumental in curbing pyroptosis in these flaps. Activation of TFE3 by FFA promoted autophagy and diminished oxidative damage. The therapeutic effects of FFA were negated when TFE3 levels were decreased using adeno-associated virus (AAV)-TFE3shRNA. Additionally, FFA modified TFE3 activity through the AMPK-TRPML1-Calcineurin pathway.

**Conclusions:**

FFA promotes ischemic flap survival via induction of autophagy and suppression of OS by activation of the AMPK-TRPML1-Calcineurin-TFE3 signaling pathway. These findings could have therapeutic implications.

## Graphical Abstract

**Graphical Abstract:**
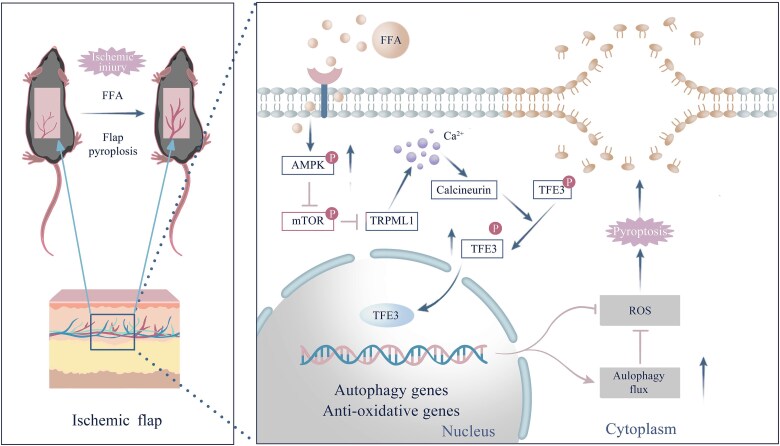

## Highlights

- Flufenamic acid (FFA) enhances autophagy and reduces OS via TFE3, thereby inhibiting pyroptosis.
- FFA modulates TFE3 via the AMPK-TRPML1-Calcineurin pathway.
- FFA is one potential medication as a treatment for ischemic flaps.

## Background

In plastic and reconstructive surgery, skin flap transplantation is commonly used to repair large-area wounds, such as severe burns and persistent ulcers [1]. However, the lack of a stable blood supply and the presence of variables such as oxidative stress (OS) and inflammation often limit its practical application, contributing to ischemic necrosis at the distal ends of flaps [2]. Random-pattern skin flaps, known for their practicality, are frequently used in studies of ischemic flaps [3]. The primary cause of distal ischemic necrosis is the infiltration of inflammation, accumulation of oxygen free radicals, and insufficient angiogenesis [4]. Creating a pro-angiogenic environment by inhibiting inflammation and OS can significantly enhance the survival of ischemic flaps, making the discovery of new methods to improve flap survival crucially important both clinically and scientifically [5].

Recent research in programmed cell death has markedly progressed, introducing novel therapeutic strategies for ischemic flaps. Previous studies focused extensively on two types of programmed cell death: apoptosis and necrosis [6]. However, attention has recently shifted to pyroptosis, a proinflammatory type of cell death affecting multiple tissues, including flaps [7]. Three key components of NLRP3 inflammasomes are Caspase-1, ASC, and NLRP3 [8]. Pyroptosis results from the inflammasome complex’s activation of IL-1β and GSDMD-N [9]. Traditionally, necrosis was considered an uncontrolled and unpredictable process, commonly occurring post-flap surgeries [10]. The recognition of pyroptosis has revised our understanding of cell death and identified potential targets for mitigating unintended cell death.

Ischemic damage predominantly generates reactive oxygen species (ROS), including the superoxide anion and singlet oxygen [11]. Furthermore, ROS play a crucial role in triggering pyroptosis [12]. Given that ROS mediate the effects of hypoxia on cellular demise, strategies that lower ROS levels or obstruct their production are pivotal in protecting cells under hypoxic conditions [13]. Therefore, boosting the synthesis of enzymes that counteract OS could markedly improve the survival prospects of ischemic flaps.

Autophagy, recognized as a vital survival mechanism in eukaryotic cells, has garnered extensive interest in scientific research [14]. It functions in conjunction with lysosomes to remove cellular debris and toxins under adverse conditions, thereby supplying essential materials and energy for cellular repair and homeostasis [15]. Previous research has demonstrated that autophagy mitigates programmed cell death by eliminating critical molecules involved in this process [16]. Therefore, modulating autophagy and programmed cell death presents a viable approach to enhancing the survival of cells and tissues.

FFA, a widely recognized nonsteroidal anti-inflammatory drug (NSAID), is already employed in clinical settings to alleviate inflammation [17]. It proved effective in suppressing IL-1β, IL-2, MCP-1, and TNF-α in bronchial asthma studies [18]. In Alzheimer’s disease, FFA successfully inhibited NLRP3 inflammasome activation via AMPK regulation [19]. Interestingly, in angiogenesis studies, FFA activated AMPK, promoting tube formation in human umbilical vein endothelial cells (HUVECs) and macroscopic vessel formation in chick chorionic allantoic membrane experiments [20]. The comprehensive functional advantages and safety of FFA suggest its potential application value in the management of ischemic flaps. Crucially, recent studies reveal that the AMPK-TRPML1-Calcineurin pathway plays a regulatory role in controlling TFE3 to manage autophagy [21]. This study explored the molecular basis and protective effects of FFA on ischemic flaps.

## Methods

### Ischemic flaps model

Due to estrogen’s known impact on flap necrosis, only male mice were utilized in these studies [22]. The protocols were approved by the Animal Care and Use Committee at Wenzhou Medical University (Approval no. wydw2024–0283). The mice were mature male C57BL/6 J, weighing 20–30 g, and they were 6–8 weeks old. A 12-hour day/night cycle, temperatures between 21 and 25°C, humidity between 50% and 60%, and unrestricted availability to food and drink were all features of the typical living environment. For the purpose of constructing the models, the mice were numbed with an intraperitoneal injection of 1% pentobarbital sodium (50 mg/kg). To get rid of the undercoat, we utilized an electric shaver and depilatory lotion. Afterwards, a 1.5 × 4.5 cm^2^ skin/panniculus carnosus flap was affixed to the mouse’s back once all visible blood vessels had been removed. Sewing the flaps back into place was then done using 4–0 nonabsorbable silk. Postoperatively, penicillin was administered intramuscularly daily to prevent infection, and iodophor was used for disinfection. This model simulated ischemic flap injury characteristics in humans. Blinded researchers conducted and assessed all animal experiments, with random allocation of mice to treatment groups.

Animal treatment (a): To establish a dose–response curve, 30 C57BL/6 J mice were prepared individually. Six groups of five mice each were randomly assigned to a dosage (mg kg^−1^ day^−1^; 0, 3, 6, 12, 24, 48). (b): The knockdown of TFE3 in mouse flaps was achieved two weeks before surgery by injecting AAV-expressing TFE3 shRNA (AAV-shTFE3) into the tail veins. An AAV-scramble negative control sequence was administered to the control group in an equal volume of AAV vehicle. Fifteen treatment groups were formed, including Control (n = 40); FFA (n = 40); ATN-224 (n = 10); FFA/ATN-224 (n = 10); 3MA (n = 15); FFA/3MA (n = 15); TFE3 shRNA (n = 20); FFA/scrambled shRNA (n = 20); FFA/TFE3 shRNA (n = 20); CC (n = 5); FFA/CC (n = 5); ML-SI1 (n = 5); FFA/ML-SI1 (n = 5); Tacrolimus (n = 5); FFA/Tacrolimus (n = 5). Following surgery, patients in the FFA group were given intraperitoneal injections of 12 mg kg^−1^ day^−1^ of FFA, whereas those in the control group were given saline. Injectable 3MA (15 mg kg^−1^), ATN-224 (28 mg kg^−1^), CC (1.5 mg kg^−1^), ML-SI1 (1 mg kg^−1^), and Tacrolimus (1 mg kg^−1^) were given daily intraperitoneally prior to FFA delivery by 30 minutes. All three groups received the same treatment: FFA, FFA/scrambled shRNA, and FFA/TFE3 shRNA.

From 1:00 to 5:00 p.m., a laser doppler and camera were used to evaluate blood supply and flap survival. Pentobarbital sodium was used to re-anesthetize the animals after 7 days of therapy. There were three portions made of the ischemia flap area: proximal (area I), intermediate (area II), and distal (area III). We prepared samples for immunohistochemistry, WB, and immunofluorescence (IF) from region II, which measured 1 cm × 1 cm. All samples were taken promptly after euthanasia, washed with PBS, and either kept or processed according to the specific needs of the study.

### Materials

FFA (C_14_H_10_F_3_NO_2_; purity ≥99.93%, HY-B1221), ML-SI1 (C_23_H_26_C_l2_N_2_O_3_; purity ≥99.85%, cat# HY-134818), and Tacrolimus (C_44_H_69_NO_12_; purity ≥99.93%, cat# HY-13756) were purchased from MCE. Sigma-Aldrich supplied 3MA (C_6_H_7_N_5；_ purity >99.91%, cat# M9281). Med Chem Express provided CC (C_24_H_25_N_5_O; purity >98.14%, cat# 866405–64-3) and ATN-224 (C_10_H_28_MoN_2_O_2_S_4_; purity ≥98%, cat# HY-16074). The hematoxylin–eosin (HE) staining kit (cat# G1120) was obtained from Solarbio Science & Technology. GeneChem Chemical Technology Co., Ltd designed AAV-TFE3 shRNA (serotype 9).

### Determination of flaps survival area

On Day 7 following the surgical procedure, the observable traits of the flaps, encompassing their appearance, coloration, and hair condition, were documented. The area of flap survival was quantified using high-resolution imaging and Image-Pro Plus software (version 6.0), applying the formula: % survival = (survival area ÷ total area) × 100%.

### LDBF

Vascular network and blood supply visualization in the flaps was performed using LDBF. A tranquil, secure environment was provided to the sedated mice on the 7th day following the surgery. Blood flow and tiny veins were assessed using laser doppler technology. To evaluate LDBF, the perfusion units were determined using the moorLDI Review program (ver. 6.1), as mentioned elsewhere [23].

### Tissue section preparation

The mice were given a deep anesthetic and saline perfusion to draw out blood on the 7th day after surgery. We preserved peri-necrotic areas of ischemia flaps (1 cm × 1 cm) in 4% (w/v) paraformaldehyde for 24 hours after liver and kidney tissues were extracted. Following their dehydration in different ethanol concentrations, the tissues were embedded in paraffin, sectioned into 4 μm pieces using a microtome, and then set on gelatin-coated slides.

### Hematoxylin–eosin (HE) staining

On Day 7 post-procedure, anesthetized mice underwent tissue sampling for histopathological examination from region II of the flaps. Six samples per group (1 cm × 1 cm) were processed. After saline perfusion to remove blood cells, samples were sectioned transversally at 4 μm, fixed in 4% paraformaldehyde for 24 hours, embedded in paraffin, and stained with HE. Histological changes were assessed under a bright-field microscope, and mean vessel density per unit area (mm^−2^) was calculated to gauge microcirculation.

### Immunofluorescence staining

Preparation of flap pieces followed the methodology outlined earlier. Once the tissues had been dewaxed and rehydrated, they were heated to 95°C and placed in a 10.2 mM sodium citrate buffer for 20 minutes. Prior to blocking with 10% (v/v) goat serum in PBS for one hour, tissues were permeabilized with 0.1% (v/v) PBS-Triton X-100 for 10 minutes. Prior to adding primary antibodies, incubate overnight at 4°C: The following sources were used: TFE3 (1:200, Sigma-Aldrich cat# HPA023881, RRID:AB_1857931), EMCN (1:200, cat# ab106100) from Proteintech Group (Chicago, IL, USA), Caspase-1 (1:200, cat# 22915–1-AP, RRID:AB_2876874) from Abcam (Cambridge, UK), LC3 (1:200, cat# 3868, RRID:AB_2137707) from Cell Signaling Technology (Beverly, MA, USA), and CD31 (1:200, cat# GB12063, RRID:AB_2941868) from Servicebio (Wuhan, China). After primary antibodies were incubated, secondary antibodies were added and incubated for another hour at 37°C. Tissues were then stained using DAPI solution (cat# ab104139, Abcam, UK). Abcam of Cambridge, UK supplied the secondary antibodies (cat# ab150113, RRID:AB_2576208) and (cat# ab150080, RRID:AB_2650602) against goat anti-rabbit IgG H&L (Alexa Fluor® 594).

### IF quantification

An imaging system developed by Zeiss, Germany, was used to capture images of specimens stained with CD31, EMCN, Caspase-1, and GSDMD-N. We used Zeiss’ Zen Blue program for image capture and processing, and we analyzed the images with ImageJ (Version 1.52a). Fluorescence microscopy was performed on specimens that had been stained with LC3 and TFE3 using a Japanese Olympus instrument. The dermis of the flaps was photographed, and five randomly selected locations from three randomly selected sections per animal were imaged inside the peri-necrotic area. As a result, we used ImageJ to determine the GSDMD-N, caspase-1, LC3, and dihydroethidium (DHE) integrated densities. Under a controlled environment, we manually determined the number of CD31-EMCN positive vessels per mm^2^. Another metric that was computed using ImageJ was the TFE3 integrated density.

### DHE staining

The tissues in flap area II were dehydrated by submerging them in a 30% sucrose solution. Once the tissues were dry, they were placed on new filter paper to soak up any remaining water. Next, they were covered in OCT and kept in the fridge until they solidified. The tissues were then cut into slices that were 20 μm thick. Slicing was followed by 5 minutes of autofluorescence quenching. After diluting the DHE stock solution with PBS to the appropriate concentration, the tissue samples were left to incubate for 30 minutes at 37°C in this solution. The last step was to photograph the tissue slides using a fluorescence microscope after three rounds of washing with PBS, each lasting 5 minutes.

### 5-FAM-conjugated collagen hybridizing peptide staining

5-FAM-conjugated collagen hybridizing peptide (F-CHP) staining was employed to detect collagen degeneration [24]. 3Helix Inc. supplied the F-CHP (cat# FLU300, FLU60). We diluted the F-CHP solution with PBS after dewaxing and rehydrating. This solution was subjected to a rapid cooling process in an ice bath for 90 s after being heated for five minutes at 80°C in a water bath to prevent heat damage. For 12 hours, the tissue samples were placed in an incubator set at 4°C. Using a Zeiss LSM 800 confocal microscope, images were taken after three room temperature rinses with PBS, following staining.

### TdT-mediated dUTP Nick end labeling staining

Tissue slices were stained with TdT-mediated dUTP Nick end labeling (TUNEL) using a Detection Kit of *in situ* Cell Death, Fluorescein (Millipore Sigma). In order to identify cells that had positive staining, a Zeiss LSM 800 confocal microscope was employed. Everything was accomplished with the help of Zeiss’ Zen Blue software for taking and processing images, and ImageJ software (Version 1.52a) for viewing the results.

### Propidium iodide staining

To stain the tissue slices, we used a propidium iodide (PI) dye solution from Servicebio in China. To find cells with positive staining, researchers utilized a Zeiss LSM 800 confocal microscope. Visualization was accomplished with the help of ImageJ (Version 1.52a).

### Nuclear and cytoplasmic fractionation

To homogenize the 80 milligrams of freshly harvested flap tissue, we utilized an ice-filled Dounce tissue grinder. The nuclear fractions were separated from the homogenates for differential centrifugation. For this mission, we relied on the NE-PERTM Nuclear and Cytoplasmic Extraction Reagents (cat# 78835, Thermo Fisher Scientific, USA). The supernatants were used to collect the cytosolic fractions. We froze the nuclear fractions at −80°C to make them easier to use in future studies.

### WB

A combination of protease and phosphatase inhibitors from Sigma-Aldrich were used to homogenize five millimeters squared of tissue from flap area II in an ice-cold RIPA lysis solution from Beyotime. The homogenates were centrifuged at 12000 rpm for 30 minutes at 4°C to obtain the tissue lysates. Thermo Fisher Scientific’s Pierce BCA protein assay kit was utilized for the purpose of determining the protein concentrations. Millipore PVDF membranes were used to transfer proteins (30 μg each sample) that had been resolved on 4%–20% SDS-PAGE gels. After blocking in 5% skim milk, the membranes were probed overnight at 4°C with the following primary antibodies: p-AMPK, p-mTOR, AMPK, Beclin1, Endothelial nitric oxide synthase (eNOS), LC3, Mammalian target of rapamycin (mTOR), NLRP3, p-AMPK, and p-TFE3(Cell Signaling Technology, USA); GAPDH, Caspase-1, Cathepsin D (CTSD), Heme oxygenase 1 (HO-1), Histone-H3, and SOD1 (Proteintech Group, USA); VEGFA, SQSTM1/p62, and TRPML1/MG-2 (Abcam, Cambridge, UK); Calcineurin and IL-1β (ABclonal Technologies, Cambridge, MA, USA); GSDMD-N and IL-18 (Affinity Biosciences, OH, USA); TFE3 (Sigma-Aldrich Chemical Company, USA); Adherin 5 (CDH5); and MMP9 (HuaBio). Next, the membranes were left at room temperature for 2 hours to incubate with HRP-conjugated IgG secondary antibodies (Bioworld Technology, Minneapolis, MN, USA). Epizyme Biomedical Technology’s Omni-ECLTM Femto Light Chemiluminescence Kit and Bio-Rad’s ChemiDocTM XRS+ Imaging System were utilized for imaging. The band intensities were measured with the help of Bio-Rad’s Image Lab 3.0 program.

### Molecular docking study

The target protein AMPK’s molecular structure (PDB ID: 4ZHX) was retrieved from the PDB database. The PubChem database was used to download the FFA structural files. The acquired structures of the target proteins were subjected to processing with the PyMOL 2.3.0 program in order to exclude water molecules and the initial ligand. In order to obtain the optimal conformation that minimizes energy, the molecular optimization of the small molecules was carried out using Chem3D software. With the help of AutoDock Tools 1.5.6, which created pdbqt files, the target proteins were prepared to be used in molecular simulation and docking with AutoDock Vina v.1.2.0. AutoDock Vina use semi-flexible docking techniques and a Lamarckian genetic strategy.

### Cell culture

A company called Procell Life Science & Technology Co., Ltd supplied the HUVECs (CL-0122). An incubator was used to cultivate the cells, with 5% CO2 and 95% air maintained at 37°C. This experiment made use of DMEM (C11995500BT) from Gibco, together with 10% sterile FBS (10099141C) and 1% penicillin–streptomycin (Gibco, 1 719 675).

### Cellular thermal shift assay

After sowing HUVECs in 100 mm dishes, the next day they were exposed to an FBS-free media containing PBS or FFA for a duration of two hours in order to conduct the cellular thermal shift assay (CETSA). Harvesting, washing, and resuspending the cells in 1 ml of PBS was done after treatment. Aliquots of 90 μl were distributed into 0.2 ml PCR tubes and heated in a PCR machine at specified temperatures (37–67°C) for 3 minutes. To remove the precipitated proteins, 20 μl of RIPA buffer was added after heating, mixed well, and then centrifuged at 15 000 g for 20 minutes at 4°C. Western blot analysis was subsequently performed on the supernatant. The CETSA curve analysis was carried out using the GraphPad Prism software, developed by GraphPad Software Inc. in the USA.

### Surface plasmon resonance

GE Healthcare, USA’s Biacore 8 K device was used to measure surface plasmon resonance (SPR). Using normal amine coupling protocols, AMPK protein (20 μg/ml, pH 4.5) was attached to an s-series sensor chip (GE Healthcare, Piscataway, NJ, USA) from GE Healthcare. Immobilization ran in a buffer consisting of PBS and 1% dimethyl sulfoxide (BR100672, pH 7.2–7.4, Cytiva). The binding phase lasted 100 s at 25 degrees Celsius and was initiated after immobilization. Different doses of FFA solution were delivered at a rate of 20 μl/min. The final graph was obtained by subtracting the response from a blank sensor. The data were examined with the use of Biacore 8 K Manager software from GE Healthcare in the USA in order to determine an equilibrium dissociation constant (Kd) of 22.1 μM and fit a binding model.

### Statistical analysis

Version 22 of SPSS was used for statistical analyses. In order to reduce causes of variability, all data were standardized. To check if the data was normal, the Shapiro–Wilk test was utilized. The results of independent-sample t-tests or one-way analysis of variance (ANOVA) with subsequent post hoc tests were used to assess normally distributed data, which is shown as mean ± standard deviation (SD). Data that did not follow a normal distribution were examined using the appropriate Mann–Whitney U or Kruskal-Wallis test and are presented as medians with interquartile ranges. ^*^P < .05 denoting substantially different; ns = not significant.

## Results

### F‌FA enhances survival of ischemic flaps

**Figure 1:**
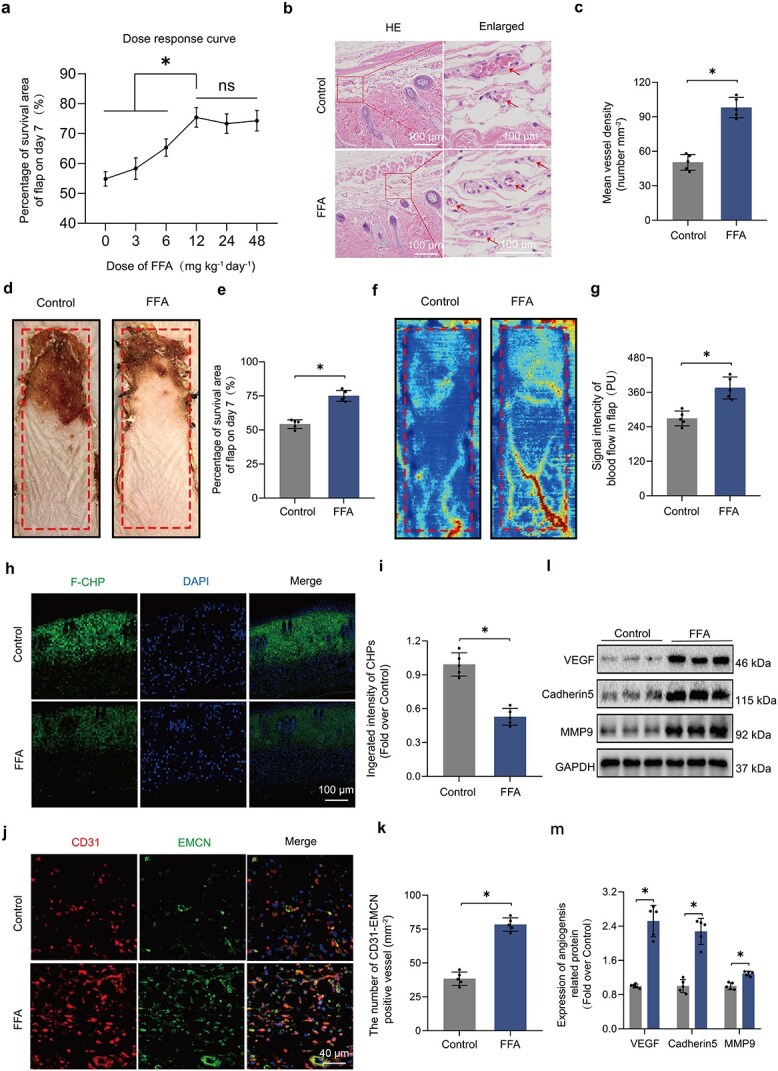
FFA enhances survival of ischemic flaps. (**a**) The dose–response curve demonstrates the optimal FFA dosage (12 mg kg^−1^ day^−1^) for a 7-day treatment period to evaluate flap survival. (**b–c**) HE staining samples from FFA and control groups in the peri-necrotic region reveal blood vessel density (mm^−2^). (**d–e**) On Day 7 post-surgery, the necrotic area was imaged, and the surviving area percentage was calculated using Image-Pro Plus software. (**f–g**) On Day 7 post-surgery, subcutaneous blood flow was assessed using LDBF; FFA treatment significantly enhanced the blood flow signal. (**h–i**) The quantification graph of CHPs appears on the right; F-CHP staining determined the damaged collagen amount in flaps from four groups on Day 7 postoperatively. (**j–k**) On Day 7 postoperatively, flaps from the stated groups were stained with CD31 and EMCN via IF. Vessel counts positive for CD31 were conducted (n = 5). (**l–m**) WB analysis of mouse flaps showing VEGF, Cadherin5, and MMP9 expression with GAPDH as the reference. Density quantification is displayed below. Two-tailed, unpaired t tests were conducted and the data are presented as the means ± SD, ^*^P < .05 denoting substantially different; ns = not significant. FFA: Flufenamic acid, HE: Hematoxylin–eosin, LDBF: Laser doppler blood flow, F-CHP:5-FAM collagen hybridizing peptide, EMCN: Endomucin, IF: immunofluorescence, WB: western blotting, VEGF: vascular endothelial growth factor, MMP9: matrix metallopeptidase 9

The optimal dose of FFA was identified as 12 mg kg^−1^ day^−1^ after assessing flap viability across various dosages (Figure 1a). HE staining was performed on samples from the necrotic connections of the flaps (Figure 1b). The examination of vascularization in necrotic areas revealed a significant enhancement in the group treated with FFA (Figure 1c). The research utilized an adapted McFarlane flap model on the dorsal regions of laboratory mice. Necrosis was initially noted at the distal ends of the flaps 3 days after the surgical procedure, progressing towards the pedicle and reaching a state of stabilization by the 7th day (Figure 1d). The FFA-treated group exhibited a significant improvement in flap survival (Figure 1e). Variations in blood flow strength within the flaps were documented using laser Doppler imaging, revealing heightened circulation in the FFA group (Figure 1f–g). Furthermore, the degradation of collagen was significantly reduced in the FFA group, as demonstrated by F-CHP staining (Figure 1h–i). The FFA group exhibited a heightened occurrence of CD31-EMCN positive vessels, highlighting its significance in promoting vascularization after ischemic injury (Figure 1j–k). Increased concentrations of angiogenesis markers, including VEGF, CDH5, and MMP9, were markedly elevated in the FFA group (Figure 1l-m). This observation indicates the possible clinical relevance of FFA in the management of ischemic flaps and necessitates further investigation to elucidate the mechanisms by which FFA enhances flap viability following ischemic damage.

### F‌FA inhibits pyroptosis in ischemic flaps

**Figure 2:**
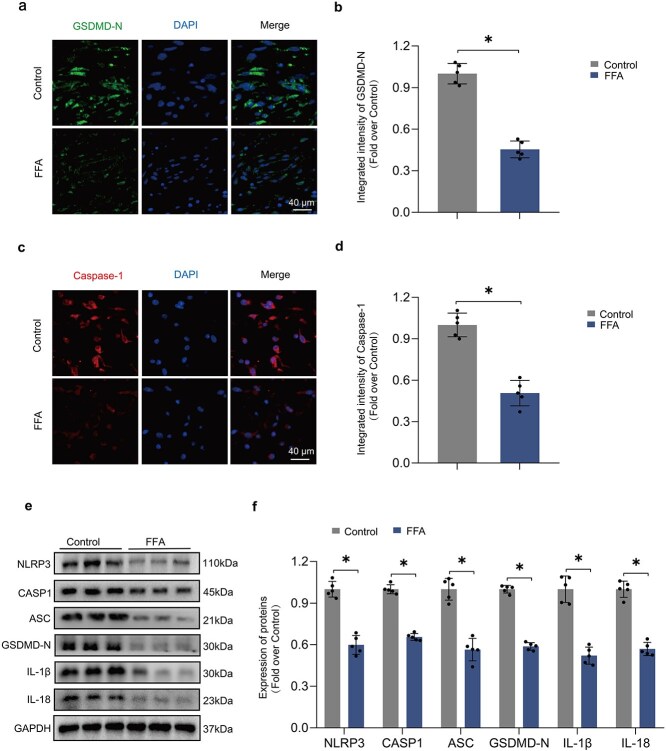
FFA inhibits pyroptosis in ischemic flaps. (**a–d**) Flap sections were stained with anti-GSDMD-N (green) or -CASP1 (red) IF antibodies. Nuclei were labeled with DAPI (blue). The adjacent graph quantifies anti-GSDMD-N (green) or -CASP1 (red) IF. (**e–f**) Pyroptosis-associated protein levels in the peri-necrotic region of ischemic flaps were measured using WB. Two-tailed, unpaired t tests were conducted and the data are presented as the means ± SD, ^*^P < .05 denoting substantially different; ns = not significant. FFA: Flufenamic acid, GSDMD: Gasdermin, CASP1: Caspase 1, IF: immunofluorescence, WB: western blotting

Pyroptosis has been increasingly recognized due to its strong link to inflammation. To investigate FFA’s effect on pyroptosis, levels of related proteins were analyzed. The investigation examined proteins associated with pyroptosis, such as ASC and GSDMD-N. Immunofluorescence identified expressions of GSDMD-N and Caspase-1 in the dermis (Figure 2a and (c), with their levels being significantly diminished in the FFA group (Figure 2b and (d). Western blot analysis provided additional evidence that all six proteins associated with pyroptosis exhibited a significant decrease in the FFA group (Figure 2e–f), suggesting that FFA plays a crucial role in alleviating pyroptosis within the dermal tissue of ischemic flaps.

**Figure 3:**
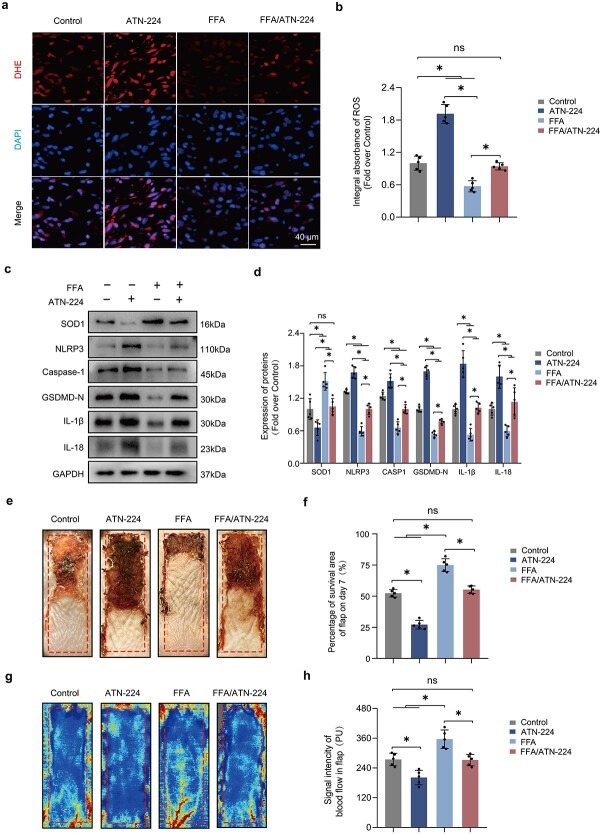
FFA mitigates pyroptosis in ischemic flaps by reducing ROS levels. (**a–b**) Sections from ATN-224, FFA, Control, and FFA/ATN-224 groups were stained with DHE (red), a ROS fluorescent probe, and nuclei were labeled with DAPI (blue). DHE IF in the dermis is quantified on the right. (**c–d**) Representative images from SOD1, NLRP3, CASP1, GSDMD-N, IL-1β, and IL-18 western blots in lysates from mouse flaps are shown. Right-side densitometry indicates that FFA partially prevented pyroptosis by inhibiting OS, an effect reversed by ATN-224, a SOD1 inhibitor. (**e–f**) A photo of the necrotic region was taken on Day 7 post-surgery. The adjacent graph quantifies the surviving area percentage and shows reduced ROS levels due to FFA, enhancing flap survival. (**g–h**) On Day 7 post-procedure, subcutaneous blood flow was assessed using LDBF, and the strength of the blood flow signal was quantified. Statistical analysis was performed using ANOVA with least significant difference post hoc tests or Dunnett’s T3 test. The data are presented as the means ± SD; ^*^P < .05 denoting substantially different; ns = not significant. FFA: Flufenamic acid, ROS: Reactive oxygen species, DHE: Dihydroethidium, IF: immunofluorescence, SOD1: Superoxide dismutase 1, NLRP3: NLR family pyrin domain containing 3, CASP1: Caspase 1, GSDMD: Gasdermin D, IL-1β: Interleukin 1β, IL-18: Interleukin 18, OS: oxidative stress, LDBF: Laser doppler blood flow

### F‌FA mitigates Pyroptosis in ischemic flaps by reducing ROS levels

Oxidative tissue damage, caused by elevated free oxygen radicals during ischemic injury, prompted an investigation into whether FFA enhances flap survival by reducing OS. OS markers were measured, and oxidative damage in ischemic flaps was assessed using DHE as a ROS probe, showing faint fluorescence in the FFA group (Figure S1a–b). SOD1, HO1, and eNOS, important enzymes that combat OS, were found to be increased in the FFA group, as demonstrated by WB (Figure S1c–d). Overall, FFA was shown to reduce OS-related damage in flaps.

The effect of ATN224 on SOD1 activity was tested next; it reversed the suppression of ROS by FFA in the dermis of ischemic flaps (Figure 3a–b). However, WB indicated that FFA’s role is multifaceted, and suppressing ROS only partially mitigates its effect on inhibiting cell death (Figure 3c–d). Further examination of flap survival and blood flow dynamics supported the premise that FFA improves flap viability by diminishing ROS levels, particularly within the dermis (Figure 3e–h).

### F‌FA enhances ischemic flap survival by promoting autophagy

The contribution of autophagy to increased flap survival due to FFA was investigated through the analysis of autophagy-related proteins. Beclin1, VPS34, LC3II, CTSD, and p62 were utilized as markers. The integrated intensity of LC3II-labeled autophagosomes was elevated in the FFA group (Figure S2a–b), and flap tissue analyses via western blot revealed decreased p62 levels and increased expressions of Beclin1, CTSD, VPS34, and LC3II in the FFA group (Figure S2c–d), demonstrating that FFA promotes autophagy in ischemic flaps.

**Figure 4:**
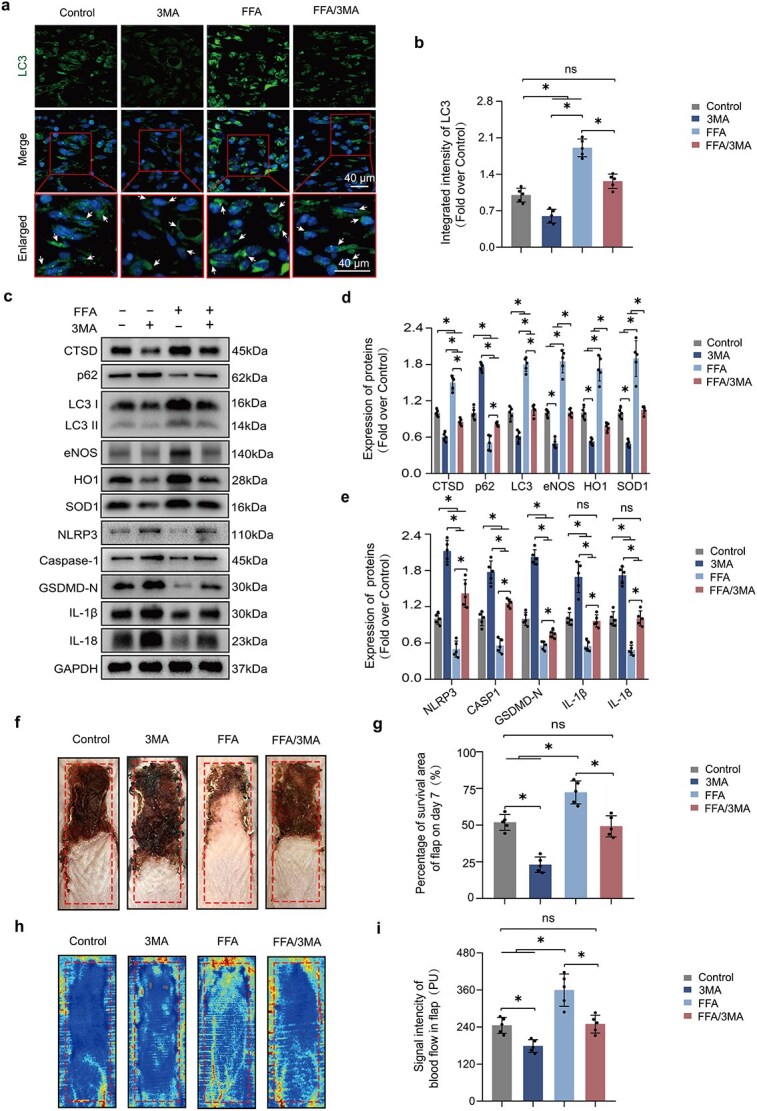
FFA enhances ischemic flap survival by promoting autophagy. (**a–b**) Flap sections from the Control, 3MA, FFA, and FFA/3MA groups were stained with anti-LC3 (green) antibodies using IF. Nuclei were labeled with DAPI (blue). The graph shows the signal quantification and includes representative images. (**c–e**) WB for CTSD, p62, LC3II, eNOS, HO1, SOD1, NLRP3, CASP1, GSDMD-N, IL-1β, and IL-18 in mouse flaps are illustrated with representative images. GAPDH was used as the reference. Graphs on the right summarize the WB data. FFA treatment enhanced flap survival by inducing autophagy; however, this effect was mitigated by 3MA’s inhibition of autophagy. (**f–g**) On Day 7 post-procedure, the necrotic region was imaged, and the graph quantified the surviving area proportion. (**h–i**) On Day 7 post-surgery, subcutaneous blood flow was measured using LDBF, and the graph quantified the strength of the blood flow signal. Statistical analysis was performed using ANOVA with least significant difference post hoc tests or Dunnett’s T3 test. The data are presented as the means ± SD; ^*^P < .05 denoting substantially different; ns = not significant. FFA: Flufenamic acid, 3MA: 3-methyladenine, WB: western blotting, CTSD: Cathepsin D, LC3: Microtubule-associated protein 1 light chain 3, eNOS: Endothelial nitric oxide synthase, HO1: Heme oxygenase 1, SOD1: Superoxide dismutase 1, NLRP3: NLR family pyrin domain containing 3, CASP1: Caspase 1, GSDMD: Gasdermin D, IL-1β: Interleukin 1β, IL-18: Interleukin 18, LDBF: Laser doppler blood flow

To elucidate the role of FFA-induced autophagy flux on flap viability, 3MA, an autophagy inhibitor, was combined with FFA to obstruct autophagy activation. This combination led to a notable reduction in the proportion of cells with LC3II-labeled autophagosomes in the FFA/3MA group (Figure 4a–b). WB analysis revealed increased p62 levels and reduced expressions of LC3II and CTSD in the FFA/3MA group (Figure 4c–d). These findings confirm that 3MA effectively blocks FFA-induced autophagy in ischemic flaps. Despite partially mitigating the anti-pyroptotic effects of FFA, inhibition of autophagy led to an increase in oxidative damage, emphasizing the role of autophagy in reducing ROS (Figure 4c–e and Figure S3a–b). Additionally, the results showed that flap survival in the FFA/3MA group was considerably lower than in the FFA group (Figure 4f–g), and laser Doppler blood flow measurements showed a significant decrease in circulation strength within the flaps in the 3MA group (Figure 4h–i).

### F‌FA promotes autophagy and reduces ROS levels through upregulation of TFE3 activity

FFA regulates autophagy and ROS levels, but the mechanisms of control over OS and autophagy are not well understood. Previous research indicates TFE3 is vital for lysosome production, autophagy, and preventing OS. To assess the impact of FFA on TFE3 regulation, the levels of TFE3 in both the cytoplasm and nucleus were analyzed. Following FFA administration, a notable increase in the nuclear levels of TFE3 within the dermal layers was evident (Figure S4a–b). Western blot analysis further revealed an elevation in TFE3 levels in both cellular compartments in the FFA-treated group (Figure S4c–f), indicating that FFA effectively enhances TFE3 levels.

**Figure 5:**
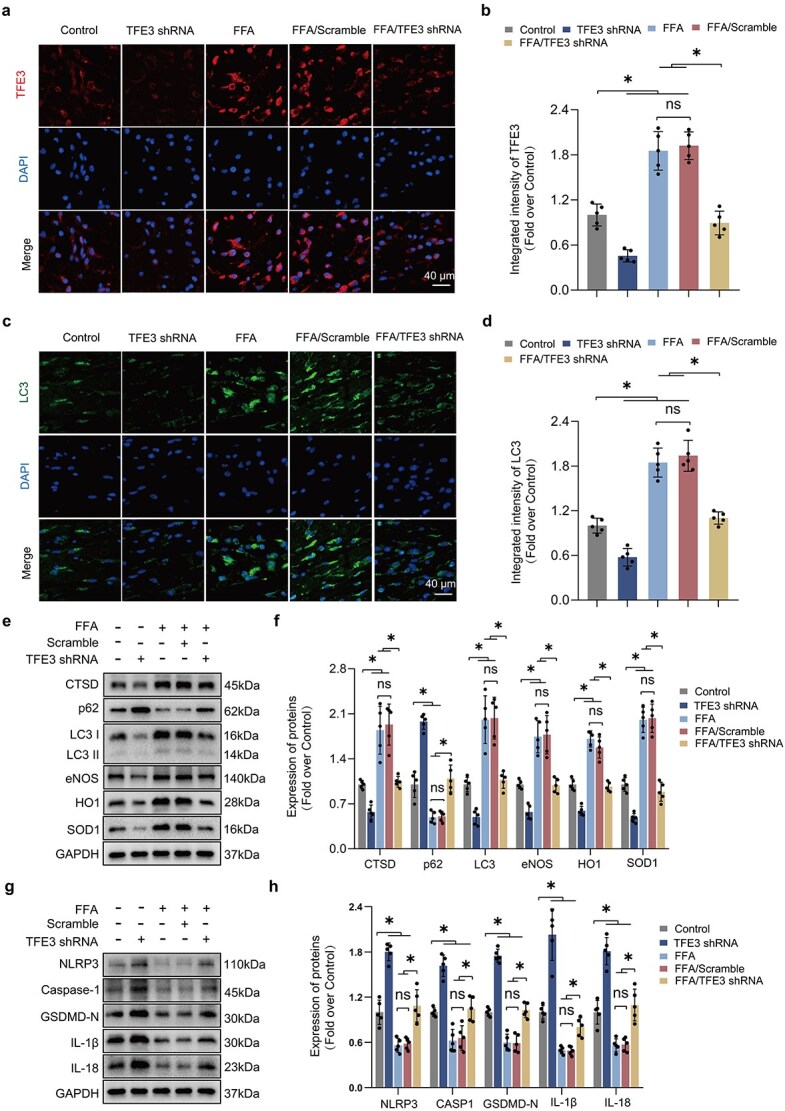
FFA promotes autophagy and reduces ROS levels through upregulation of TFE3 activity. (**a–b**) Representative IF images show nuclear TFE3 (red) levels in the peri-necrotic regions of flaps. Quantification is displayed on the graph. Flap specimens were taken from mice in the Control, TFE3 shRNA, FFA, FFA/AAV-scramble, and FFA/TFE3 shRNA groups. (**c–d**) IF staining of flaps from the mentioned groups is shown alongside a quantification graph of LC3 (green) integrated intensity. Nuclei were stained with DAPI (blue). (**e–h**) WB assesses protein levels related to autophagy, anti-OS, and pyroptosis in the peri-necrotic region. GAPDH served as the reference. Statistical analysis was performed using ANOVA with least significant difference post hoc tests or Dunnett’s T3 test. The data are presented as the means ± SD; ^*^P < .05 denoting substantially different; ns = not significant. FFA: Flufenamic acid, ROS: Reactive oxygen species, TFE3: Transcription factor E3, AAV: Adeno-associated virus, LC3: Microtubule-associated protein 1 light chain 3, WB: western blotting, OS: oxidative stress

To validate the role of TFE3 in enhancing autophagy and reducing OS via FFA, AAV-TFE3 shRNA was employed to inhibit TFE3 activity. In comparison to the FFA/scramble group, TFE3 levels were significantly diminished in the FFA/sh TFE3 group within both the cytoplasm and nucleus, with no significant variations observed between the FFA and FFA/scramble groups (Figure S4g–j). Immunofluorescence confirmed that TFE3 shRNA markedly reversed the increase in nuclear TFE3 induced by FFA in ischemic flaps (Figure 5a–b), demonstrating a substantial reduction in TFE3 expression due to TFE3 shRNA.

The study further investigated whether FFA elevates nuclear TFE3 to regulate autophagy and OS. IF assays detected no significant changes in the proportion of LC3-labeled autophagosomes between the FFA and FFA/scramble groups; however, a significant reduction was noted in the FFA/TFE3 shRNA group (Figure 5c–d). Western blot results consistently showed similar levels of CTSD, LC3, and p62 between the FFA and FFA/scramble groups, while the FFA/TFE3 shRNA group exhibited a rise in p62 and a decrease in LC3 expression. Additionally, this group showed markedly reduced levels of the antioxidant proteins SOD1, eNOS, and HO-1 (Figure 5e–f), along with increased levels of pyroptosis-related proteins (Figure 5g–h). These findings suggest that FFA supports autophagy and reduces OS in ischemic flaps primarily by promoting nuclear levels of TFE3.

To confirm if TFE3 nuclear levels contributes to increasing flap viability, the therapeutic effect of FFA was assessed post-transfection with TFE3 shRNA. Figure S5a–b demonstrate that the surviving region of the flaps was significantly smaller in the FFA/TFE3 shRNA group compared to the FFA and FFA/scrambled shRNA groups. LDBF results also showed a significant reduction in blood flow signal strength in the FFA/TFE3 shRNA group (Figure S5c–d). These results demonstrate that enhanced autophagy and reduced OS through TFE3 nuclear levels are key to FFA’s therapeutic effects.

### F‌FA forms a strong association with AMPK

**Figure 6:**
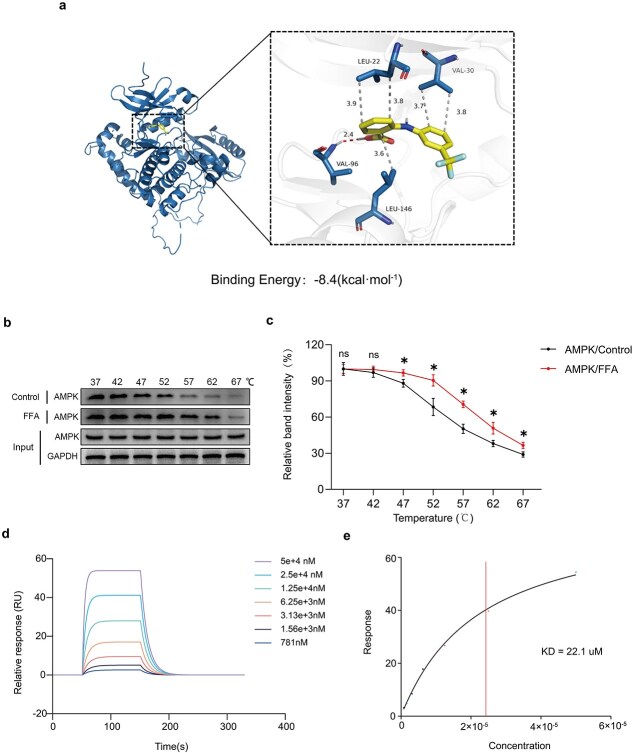
FFA forms a strong association with AMPK. Illustrations of banding patterns and a 3D binding model of FFA and AMPK complexes are displayed. (**b–c**) FFA enhanced AMPK’s thermal stability, as demonstrated by CETSA in HUVECs. Representative AMPK western blots and intensity statistics are provided on the graph. (**d–e**) Biacore analysis results for FFA and AMPK interaction are depicted. The dots represent various concentrations of FFA. FFA binds to AMPK with a KD of 22.1 μM. Two-tailed, unpaired t tests were conducted and the data are presented as the means ± SD, ^*^P < .05 denoting substantially different; ns = not significant. FFA: Flufenamic acid, AMPK: Adenosine 5′-monophosphate-activated protein kinase, CETSA: Cellular thermal shift assay, HUVECs: Human Umbilical Vein Endothelial Cells

Previous studies have shown FFA activation of AMPK [20], yet molecular docking (MD) was conducted to test FFA’s affinity for AMPK. The results were visualized using PyMOL2.3.0 software, revealing that FFA forms a strong hydrogen bond with VAL 96 at a distance of 2.4 Å and engages in multiple hydrophobic interactions with LEU 22, VAL 30, and LEU 146. The binding energy was −8.4 kcal/mol, indicating strong binding affinity to AMPK’s binding site (Figure 6a). To further investigate FFA’s interaction with AMPK, CATSA and SPR analyses were performed [25]. CATSA results indicated an increase in AMPK’s melting temperature upon FFA binding (Figure 6b–c). SPR analysis confirmed FFA’s dose-dependent interaction with AMPK, with a determined equilibrium dissociation constant (Kd) of 22.1 μM (Figure 6d–e). Thus, FFA’s binding to AMPK is highly likely to influence its pharmacodynamic effects.

### F‌FA activates TFE3 via the AMPK-TRPML1-Calcineurin pathway

Further studies seek to elucidate the mechanisms by which FFA functions. Research has uncovered that the AMPK-TRPML1-Calcineurin cascade serves as a vital calcium signaling pathway that affects the MiT/TFE family, leading to a deeper inquiry into the interaction of FFA with this signaling mechanism [26].

**Figure 7:**
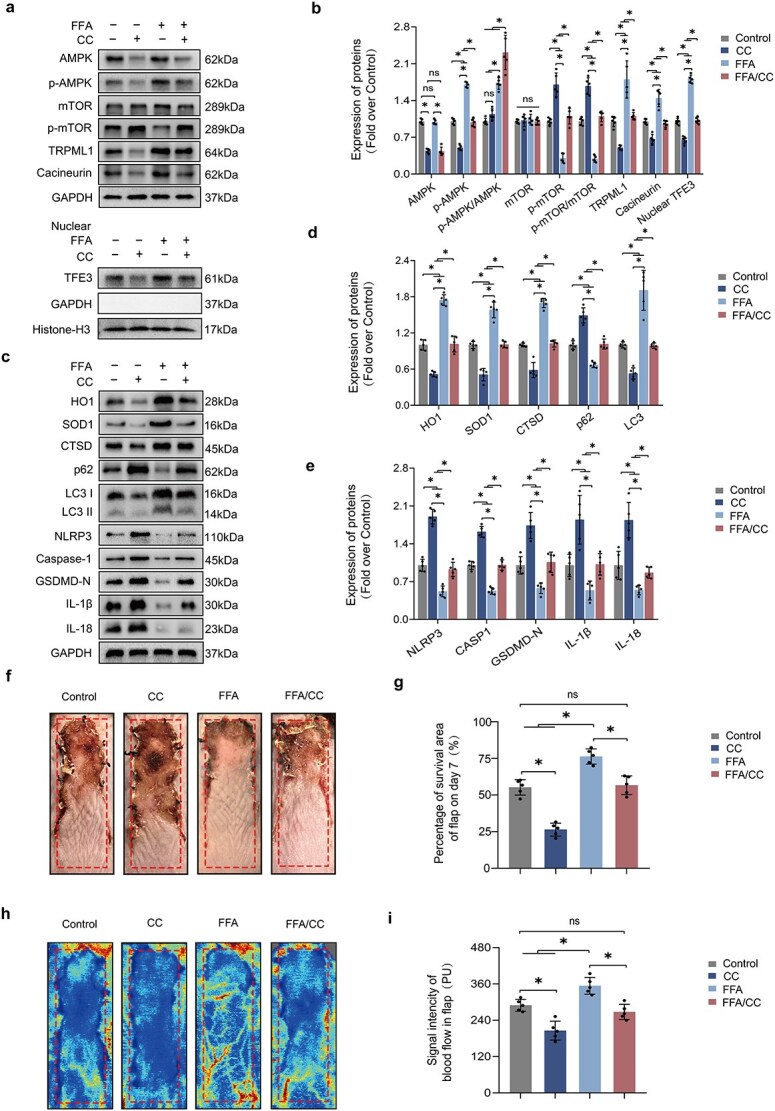
FFA activates TFE3 via the AMPK-TRPML1-Calcineurin signaling pathway. (**a–e**) Mouse tissue samples from the Control, CC, FFA, and FFA/CC groups were analyzed for levels of AMPK, p-AMPK, mTOR, p-mTOR, TRPML1, Calcineurin, nuclear TFE3, HO1, SOD1, CTSD, p62, LC3II, NLRP3, CASP1, GSDMD-N, IL-1β, and IL-18. Histone H3 or GAPDH were used as loading controls. Densitometric quantification indicated that CC, an AMPK inhibitor, attenuated the effects of FFA. (**f–g**) On Day 7 post-surgery, the necrotic region was imaged, and the graph quantified the surviving area percentage. (**h–i**) On Day 7 post-procedure, subcutaneous blood flow was measured using LDBF, and the graph quantified the strength of the blood flow signal. Statistical analysis was performed using ANOVA with least significant difference post hoc tests or Dunnett’s T3 test. The data are presented as the means ± SD; ^*^P < .05 denoting substantially different; ns = not significant. FFA: Flufenamic acid, TFE3: Transcription factor E3, CC: Compound C, AMPK: Adenosine 5′-monophosphate-activated protein kinase, mTOR: Mammalian target of rapamycin, TRPML1: Transient receptor potential mucolipin 1, HO1: Heme oxygenase 1, SOD1: Superoxide dismutase 1, CTSD: Cathepsin D, LC3: Microtubule-associated protein 1 light chain 3, NLRP3: NLR family pyrin domain containing 3, CASP1: Caspase 1, GSDMD: Gasdermin D, IL-1β: Interleukin 1β, IL-18: Interleukin 18

To determine if FFA stimulates this pathway in ischemic flaps, the expression of proteins linked to this cascade were analyzed. Techniques such as MD, CETSA, and SPR have validated the direct interaction between FFA and AMPK. The findings revealed increased concentrations of phosphorylated AMPK and decreased concentrations of phosphorylated mTOR within the cytoplasm (Figure 7a–b). The expressions of TRPML1 and calcineurin were also elevated in the FFA group (Figure 7b), confirming the activation of the AMPK-TRPML1-Calcineurin pathway by FFA.

To verify if TFE3 activation by FFA is mediated through this pathway, the effects of CC (an AMPK blocker), ML-SI1 (a TRPML1 inhibitor) [27], and Tacrolimus (a calcineurin inhibitor) [28] were assessed. These inhibitors countered FFA’s effects on TFE3 nuclear levels and activation of the signaling pathway (Figure 7a–b and Figure S6a–f). CC notably reduced FFA-induced autophagy enhancement, OS suppression, and pyroptosis reduction in ischemic flaps, as evidenced by western blot results (Figure 7c–e). Subsequent treatment with a CC led to significant reductions in both flap survival area and blood flow strength (Figure 7f–i). These observations substantiate that the AMPK-TRPML1-Calcineurin pathway facilitates the enhancement of TFE3 activity by FFA in ischemic flaps.

## Discussion

Ischemic necrosis of distal flaps is a significant complication that limits the broader clinical use of skin flap transplantation [29]. The predominant cause of distal flap necrosis is identified as ischemic damage [30], with OS being a key mechanism that either causes tissue necrosis or triggers programmed cell death [31]. Previous studies have demonstrated FFA’s role in activating AMPK, which supports angiogenesis and exhibits potent anti-inflammatory and antioxidative effects [32]. Consequently, this study investigated FFA’s therapeutic potential in ischemic flaps. Our findings indicate that FFA effectively reduces OS and pyroptosis in the dermis, significantly minimizing flap necrosis. Additionally, TFE3 activation was pinpointed as a key mechanism through which FFA exerts its therapeutic effects, markedly improving the survival of ischemic flaps.

Both apoptosis and necrosis have significantly contributed to the understanding of cell death in recent years. Interestingly, FFA has been found to inhibit both apoptosis (Figure S7) and necrosis (Figure S8) in ischemic flaps. Additionally, pyroptosis, a more recent form of programmed cell death, has garnered significant attention [33]. The primary consequence of flap surgery is often irreversible necrosis at the distal end of the flaps, which increases the patient burden and the likelihood of requiring a second procedure [34]. Previously, programmed cell death was considered unpredictable and uncontrollable [35], but the discovery of pyroptosis revealed its regulated nature [36]. Herefore, this paper focuses on the mechanisms of pyroptosis in ischemic flaps. Precise modulation of programmed cell death pathways may be necessary to prevent excessive cell death and preserve tissue viability [37]. To ascertain whether FFA prevents pyroptosis, we employed standard molecular techniques. Results demonstrated that FFA significantly lowered the levels of pyroptosis-associated proteins, thereby effectively inhibiting pyroptosis, especially in the dermal region of ischemic flaps.

**Figure 8:**
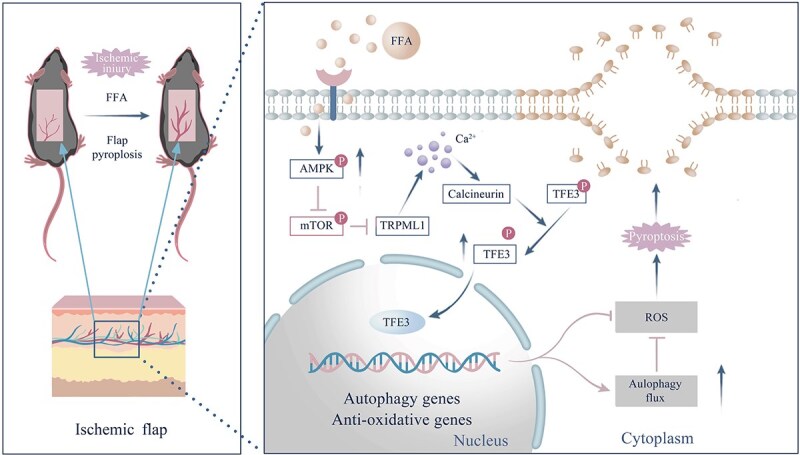
Schematic representation of the proposed molecular mechanism. The diagram depicts the roles of TFE3, ROS, pyroptosis, and autophagy in the pathophysiological processes underlying flap necrosis and their impact on enhancing flap survival. TFE3: Transcription factor E3, ROS: Reactive oxygen species

OS is crucial to the necrosis of ischemic flaps. As ROS accumulates, the pressure within tissue cells increases, leading to the progressive development of pyroptosis [38]. Our study found that FFA significantly reduced OS in the dermis. HO1, SOD1, and eNOS, which are key enzymes that combat OS, were significantly upregulated following FFA administration [39]. Moreover, it is noteworthy that the inhibition of pyroptosis by FFA was not fully reversed by reducing SOD1 activity with ATN-224. These findings suggest that an additional mechanism may contribute to FFA’s inhibitory action.

Autophagy is crucial for cell survival, relying on lysosomes and conserved through evolution [40]. To elucidate the manner in which FFA addresses ischemic flap conditions, we investigated the autophagy pathways involved. Our research measured autophagy flux and found elevated levels of autophagy-associated proteins in the dermis of mice treated with FFA. Using 3MA to inhibit autophagy increased ROS levels, decreased proteins associated with OS, and triggered pyroptosis, subsequently reducing flap vitality. Together, these results suggest that FFA enhances flap survival by inducing autophagy, which lowers ROS levels and partially prevents pyroptosis. We further investigated the upstream therapeutic mechanisms explaining how FFA enhances autophagic flux and ROS clearance in ischemic flaps. TFE3, a MiT/TFE family member, is critical for managing OS, autophagy, and lysosomal biogenesis [41]. Previous studies have shown that TFE3 activation maintains tissue function, increases levels of key enzymes such as SOD1 and eNOS, and promotes autophagic flow [42]. TFE3, under the modulation of p-mTOR [43], is activated and migrates to the nucleus to regulate gene expression associated with autophagy and oxidative resistance. In the FFA-treated group, activation of TFE3 led to an increase in proteins that promote autophagy and resistance to oxidation, a decrease in ROS levels, a suppression of pyroptosis, and an enhancement in the survival of ischemic flaps. Suppressing TFE3 expression with TFE3shRNA reversed FFA’s beneficial effects, demonstrating that FFA promotes nuclear TFE3 levels in ischemic flaps, enhancing autophagy and reducing OS, thereby effectively treating flap necrosis.

Considering the positive therapeutic effects of FFA, we also explored how FFA regulates TFE3 activity. Notably, previous research has shown that AMPK, activated by FFA in tissues, is a major regulator of the MiT/TFE family [44] and responds critically to adverse external stimuli [45]. mTOR, a serine/threonine kinase, plays a key role in regulating cell metabolism and is influenced by the AMPK-mTOR pathway [46], which stimulates cytoplasmic activities and lysosome formation through Ca^2+^ mediation by the TRPML1 channel [47]. MD, CETSA, and SPR studies confirmed that FFA binds tightly to AMPK. Furthermore, applying FFA to ischemic flaps activated the AMPK-TRPML1-Calcineurin pathway. Using CC (an AMPK blocker), ML-SI1 (a TRPML1 inhibitor), and Tacrolimus (a calcineurin inhibitor) prevented activation of these pathway-associated proteins by FFA. Overall, our results suggest that FFA elevates nuclear TFE3 levels in ischemic flaps through an upstream mechanism involving the AMPK-TRPML1-Calcineurin pathway (Figure 8).

We believe that FFA is a viable and effective treatment option crucial for managing ischemic flap necrosis. However, several issues must be resolved before clinical use in flap transplant patients. (i) FFA, as an NSAID, demonstrates therapeutic potential in managing ischemic flaps; however, further research is needed to determine its adverse effects. (ii) Ischemic damage is a common medical issue affecting vital organs such as the brain and heart. Consequently, additional research on FFA’s effects is necessary, as its therapeutic actions on flaps may extend to ischemia in other tissues.

This inquiry has several limitations that warrant further investigation. Autophagy is essential for cell viability [48]. Our research showed that FFA-stimulated TFE3-dependent autophagy had a protective effect, with an increase in the antioxidant stress protein, eNOS, observed in ischemic flaps [49]. Previous studies suggest that excessive autophagy activation may lead to detrimental outcomes [50], and autophagy is negatively regulated by the eNOS pathway [51]. This may involve eNOS’s role in regulating autophagy within safe limits to prevent excessive autophagy. Moreover, previous studies have also reported eNOS as a marker of OS [39]. Under pathological conditions, increased OS may impair eNOS function, resulting in superoxide production and further exacerbating OS [52]. This impairment is likely due to eNOS becoming dysfunctional under excessive OS, which is tightly linked to its regulatory role in maintaining OS within a protective range [53]. However, numerous studies on ischemic flaps have indicated that eNOS can inhibit OS [51–54]. Interestingly, eNOS were found to modulate vascular function and promote angiogenesis through NO/cGMP signaling pathway, and these mechanisms need further investigation [55]. It remains unclear whether eNOS directly interacts with TFE3 or modifies TFE3 expression or activity through downstream signaling pathways, thereby affecting angiogenesis and autophagy. Notably, our research highlights the critical role of ROS levels in the peri-necrotic region and FFA’s significant inhibition of pyroptosis.

We also found interesting phenomena, in our study, p-TFE3 is unfavorable to the nuclear levels and transcriptional activity of TFE3 (Figures 6a–b). This may be due to the different binding sites of p-TFE3, which lead to different corresponding regulatory mechanisms. For example, the p-TFE3 sites include S321 S567 S568 S570 [21–26]. TFE3 is homologousto TFEB, which was reported to be phosphorylated at Ser211 by mTOR [56]. The Ser211 residue(S320 in mouse TFE3; S321 in human TFE3) and the adjacent sequences are largely conserved between TFEB and TFE3 [56]. There literature also shown that the S211 phosphorylation site of TFEb negatively regulates the nuclear levels of TFEb [57], which is consistent with our study. In addition, our research results show that TFE3 levels are significantly increased after FFA treatment, which suggests that FFA treatment may promote the direct regulatory effect of AMPK on TFE3.

## Conclusions

Our study demonstrates that FFA increases nuclear TFE3 levels via the AMPK-TRPML1-Calcineurin pathway, which promotes autophagy and prevents OS in ischemic flaps. Increased autophagy partially reduces ROS levels, which further inhibits pyroptosis, particularly in the dermis. These effects enhance the survival prospects of ischemic flaps. Although FFA shows considerable potential for medical use, extensive testing and optimization are required before it can be used clinically.

## Supplementary Material

Supplementary_Information_tkaf007

## Data Availability

Upon reasonable request, the corresponding author will provide the data confirming the study’s results. Certain data might not be made public due to moral or security issues.

## Abbreviations

FFA = Flufenamic acid; LDBF = Laser doppler blood flow; AMPK = Adenosine 5′-monophosphate-activated protein kinase; TRPML1 = Transient receptor potential mucolipin 1; TFE3 = Transcription factor E3; NLRP3 = NLR family pyrin domain containing 3; ASC = Apoptosis-associated speck-like protein containing a caspase recruitment segment; CASP1 = Caspase 1; IL-1β = Interleukin 1β; GSDMD = Gasdermin D; SOD1 = Superoxide dismutase 1; AAV = Adeno-associated virus; 3MA = 3-methyladenine; CC = Compound C; HPLC = High-performance liquid chromatography; HE = Hematoxylin–eosin; TUNEL = TdT mediated dUTP Nick End Labeling; PI = Propidium iodide; LC3 = Microtubule-associated protein 1 light chain 3; eNOS = Endothelial nitric oxide synthase; mTOR = Mammalian target of rapamycin; CTSD = Cathepsin D; HO1 = Heme oxygenase 1; DHE = Dihydroethidium; EMCN = Endomucin; F-CHP = 5-FAM collagen hybridizing peptide; ROS = Reactive oxygen species; OS = oxidative stress; MD = molecular docking; CETSA = Cellular thermal shift assay; SPR = Surface plasmon resonance
