# Supplementary material for: Flufenamic acid inhibits pyroptosis in ischemic flaps via the AMPK-TRPML1-Calcineurin signaling pathway
Source: Burns Trauma. 2025 Feb 17;13:tkaf007. doi: 10.1093/burnst/tkaf007 (PMC12253954; doi:10.1093/burnst/tkaf007)
Supplement: Supplementary_Information_tkaf007 [file supplementary_information_tkaf007.docx]

**Supplementary information**

**for**

**Flufenamic acid inhibits pyroptosis in ischemic flaps via the AMPK-TRPML1-Calcineurin signaling pathway**

**Liang Chen et al.**

**Figures and figure legends**

**
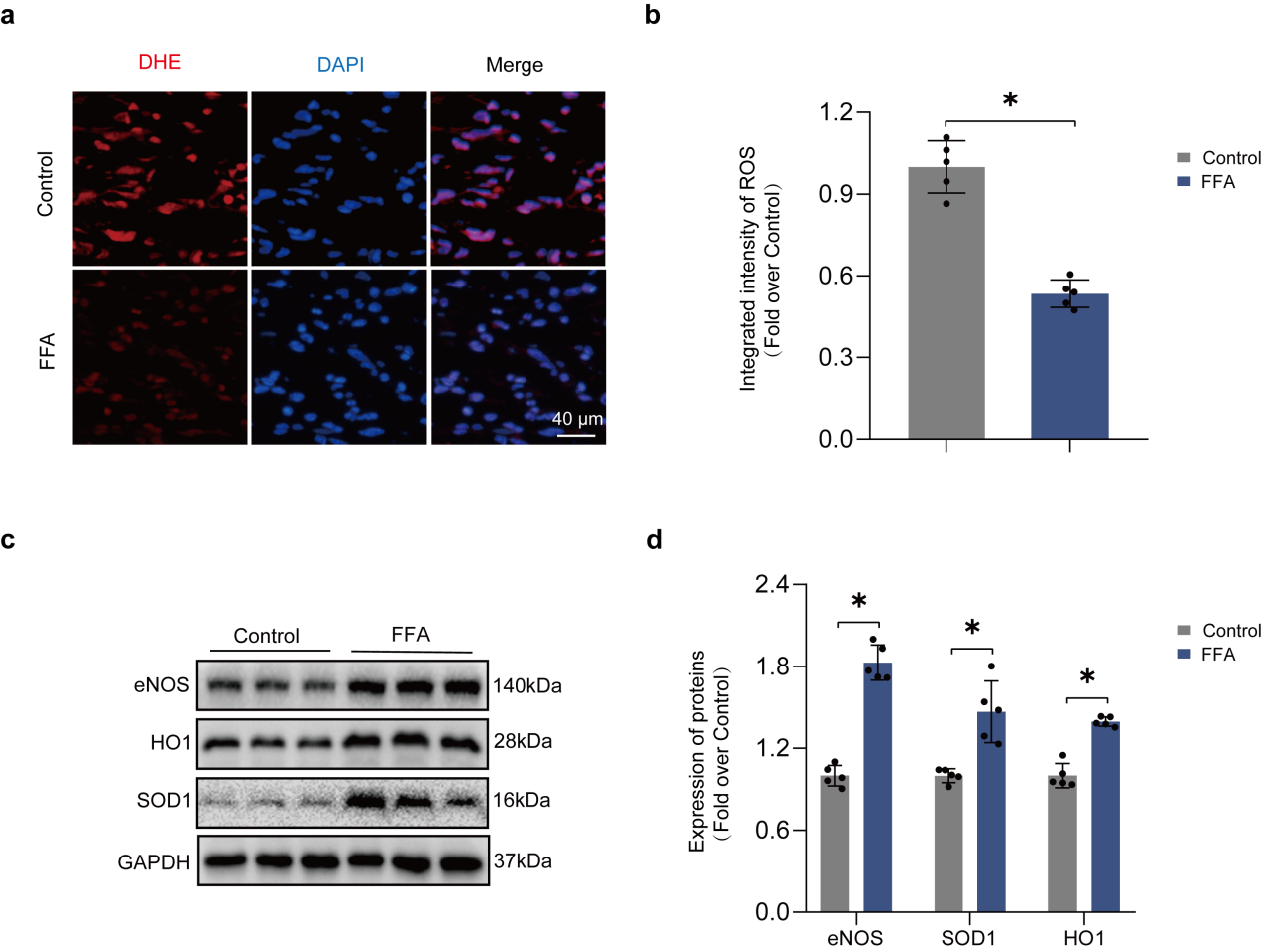
**

**Figure S1. FFA enhances ischemic flap survival by reducing ROS.**

**(a-b)** DHE (red; a ROS fluorescence probe) stained frozen sections from the Control and FFA groups. Nuclei were labeled with DAPI (blue). On the right, a quantification graph of DHE immunofluorescence is shown. **(c–d)** Western blot analysis of mouse flaps demonstrated levels of eNOS, HO1, and SOD1. GAPDH was used as the reference. Summary data from the WB are presented in the graphs on the right. FFA treatment enhanced the enzymes’ resistance to OS, thereby preserving the flaps. Two-tailed, unpaired t tests were conducted and the data are presented as the means ± SD; *p < 0.05 denoting substantially different; ns = not significant. FFA: Flufenamic acid, DHE: Dihydroethidium, eNOS: Endothelial nitric oxide synthase, HO1: Heme oxygenase 1, SOD1: Superoxide dismutase 1, OS: oxidative stress.


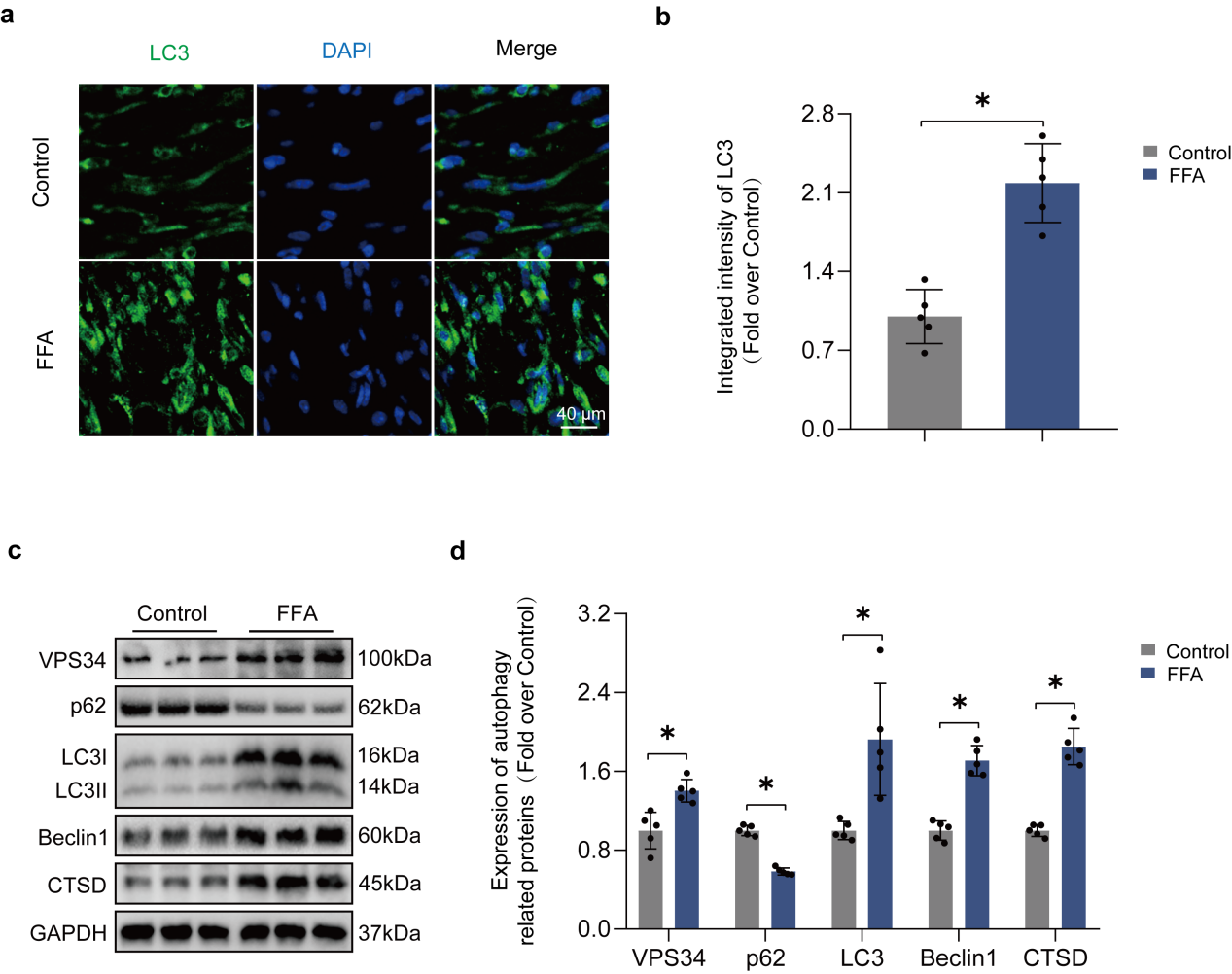


**Figure S2. FFA promotes ischemic flap survival by inducing autophagy.**

**(a–b)** LC3II (green) IF antibodies stained sections from the Control and FFA groups. Nuclei were stained with DAPI (blue). **(c–d)** Representative images from a western blot analysis of autophagy-related proteins are shown. GAPDH served as the reference. Density quantification analysis appears on the right. Two-tailed, unpaired t tests were conducted and the data are presented as the means ± SD; *p < 0.05 denoting substantially different; ns = not significant. FFA: Flufenamic acid, LC3: Microtubule-associated protein 1 light chain 3, IF: immunofluorescence.


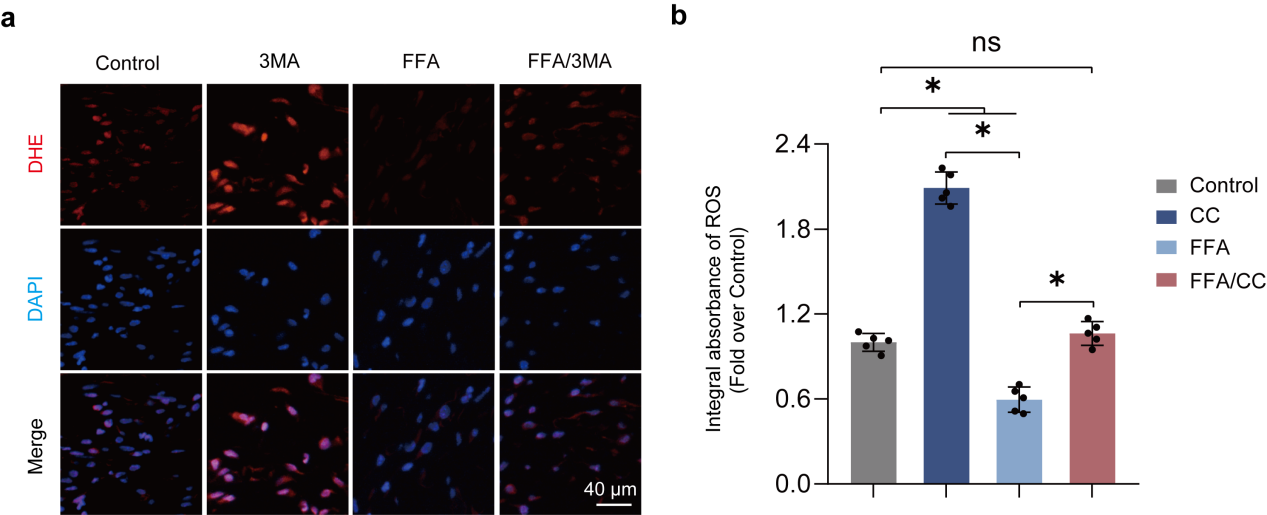


**Figure S3. FFA reduces ROS via enhanced autophagy in ischemic flaps.**

**(a–b)** Sections from each group (Control, 3MA, FFA, and FFA/3MA) were stained with DHE (red; a ROS fluorescent probe). Nuclei staining with DAPI (blue) is shown. A quantification graph of DHE IF is displayed on the right. Statistical analysis was performed using ANOVA with least significant difference post hoc tests or Dunnett’s T3 test. The data are presented as the means ± SD; *p < 0.05 denoting substantially different; ns = not significant. FFA:Flufenamic acid, 3MA:3-methyladenine, DHE: Dihydroethidium, ROS: Reactive oxygen species, IF: immunofluorescence.


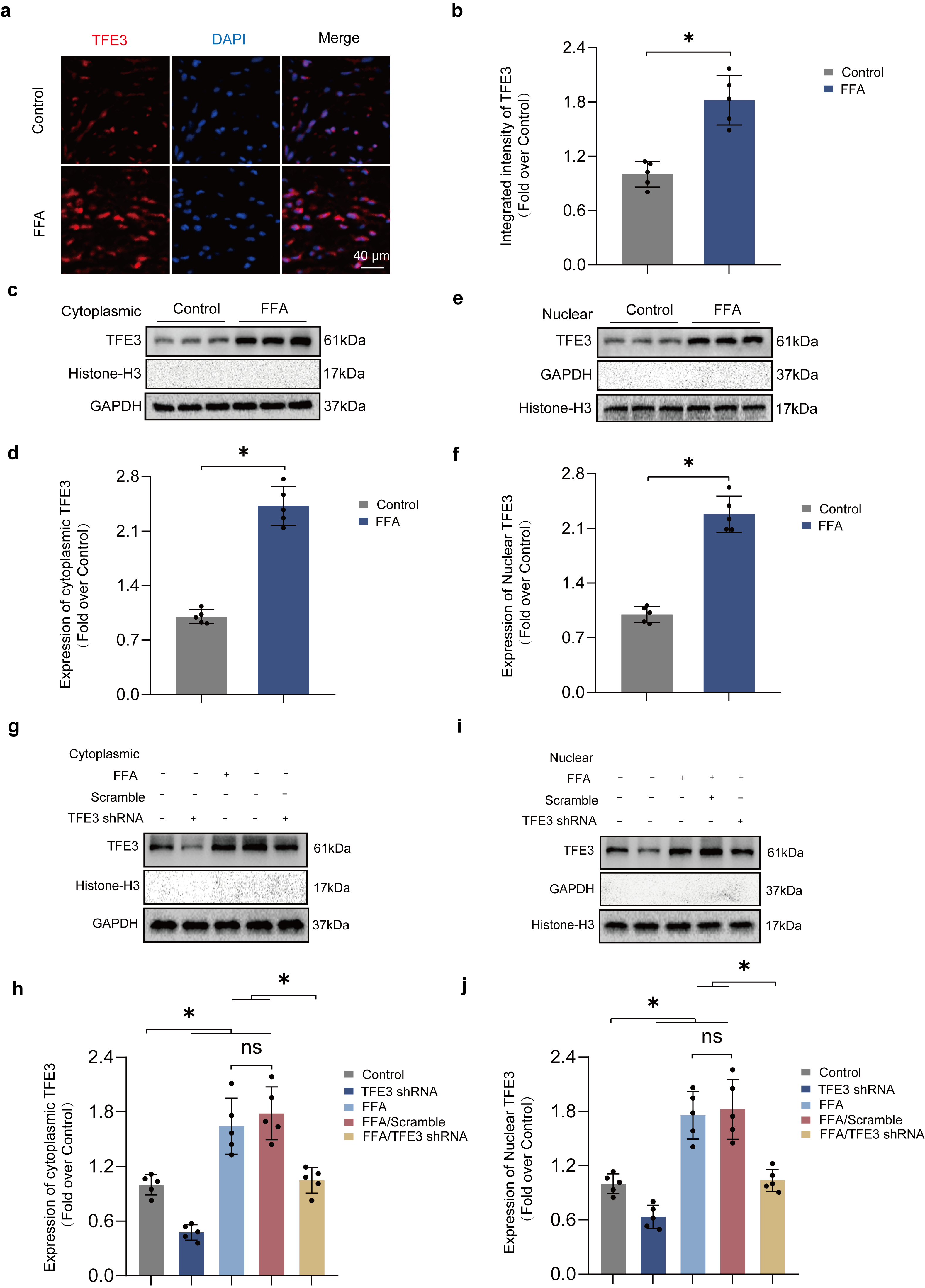


**Figure S4. Nuclear TFE3 levels Are inhibited by TFE3 shRNA After FFA treatment in ischemic flaps.**

1. Illustrative IF images show the location and levels of TFE3 (red) in the peri-necrotic region (scale bar = 40 μm). **(b)** Nuclear TFE3 levels from the images in A were quantified. **(c–j)** WB analysis examined TFE3 levels in the cytoplasm and nucleus of mouse flaps. Histone H3 or GAPDH was used as the loading control. Two-tailed, unpaired t tests were conducted and the data are presented as the means ± SD **（a-f）**;statistical analysis was performed using ANOVA with least significant difference post hoc tests or Dunnett’s T3 test. The data are presented as the means ± SD (g-j); *p < 0.05 denoting substantially different; ns = not significant. TFE3: Transcription factor E3, FFA: Flufenamic acid, IF: immunofluorescence, WB: western blotting.


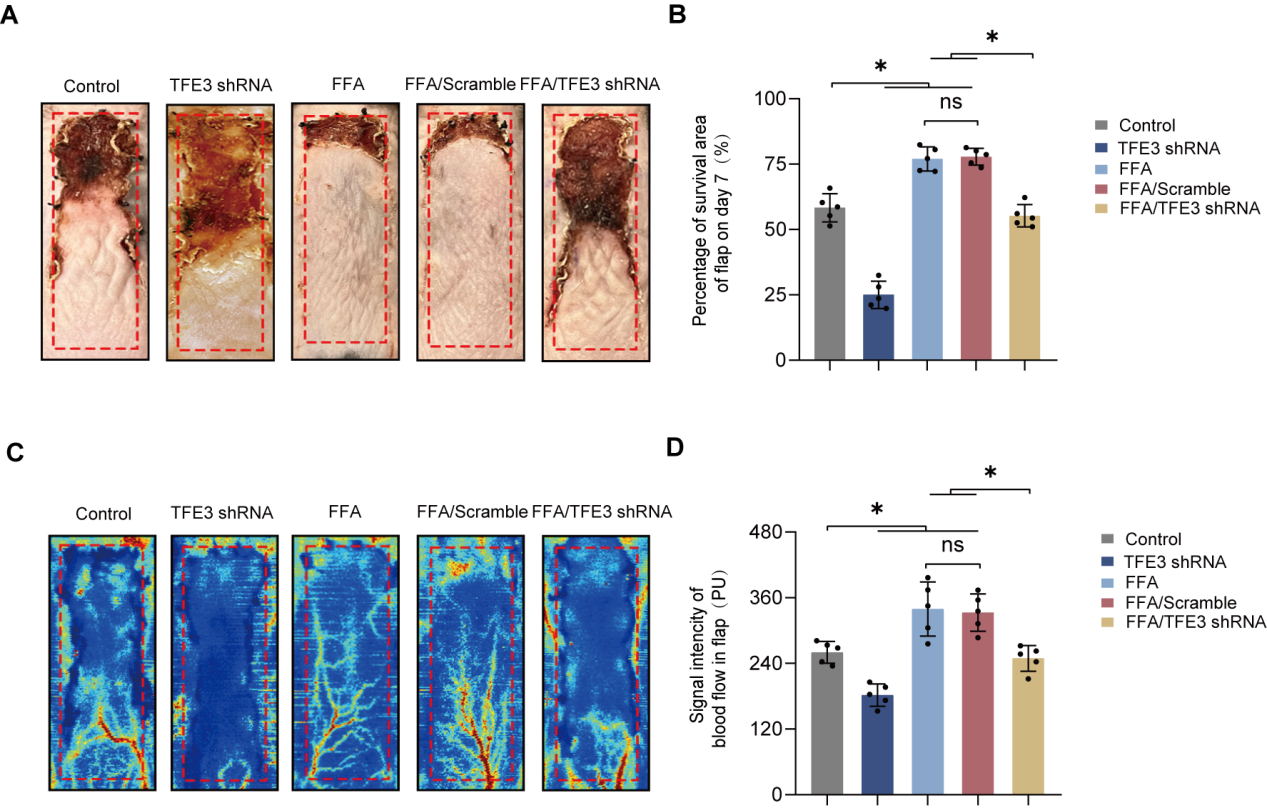


**Figure S5. FFA enhances ischemic flap survival by upregulating TFE3 activity.**

**(a-b)** On day 7 post-surgery, digital images of the necrotic region were captured, and the surviving area proportion was determined using Image-Pro Plus software. Intraperitoneal administration of FFA (12 mg kg^-1^ day^-1^) significantly improved flap survival, although TFE3 shRNA mitigated FFA’s effects. **(c–d)** On day 7 post-surgery, subcutaneous blood flow was measured using LDBF, with a graph quantifying the blood flow signal strength. Statistical analysis was performed using ANOVA with least significant difference post hoc tests or Dunnett’s T3 test. The data are presented as the means ± SD; *p < 0.05 denoting substantially different; ns = not significant. FFA: Flufenamic acid, TFE3: Transcription factor E3, LDBF: Laser doppler blood flow.


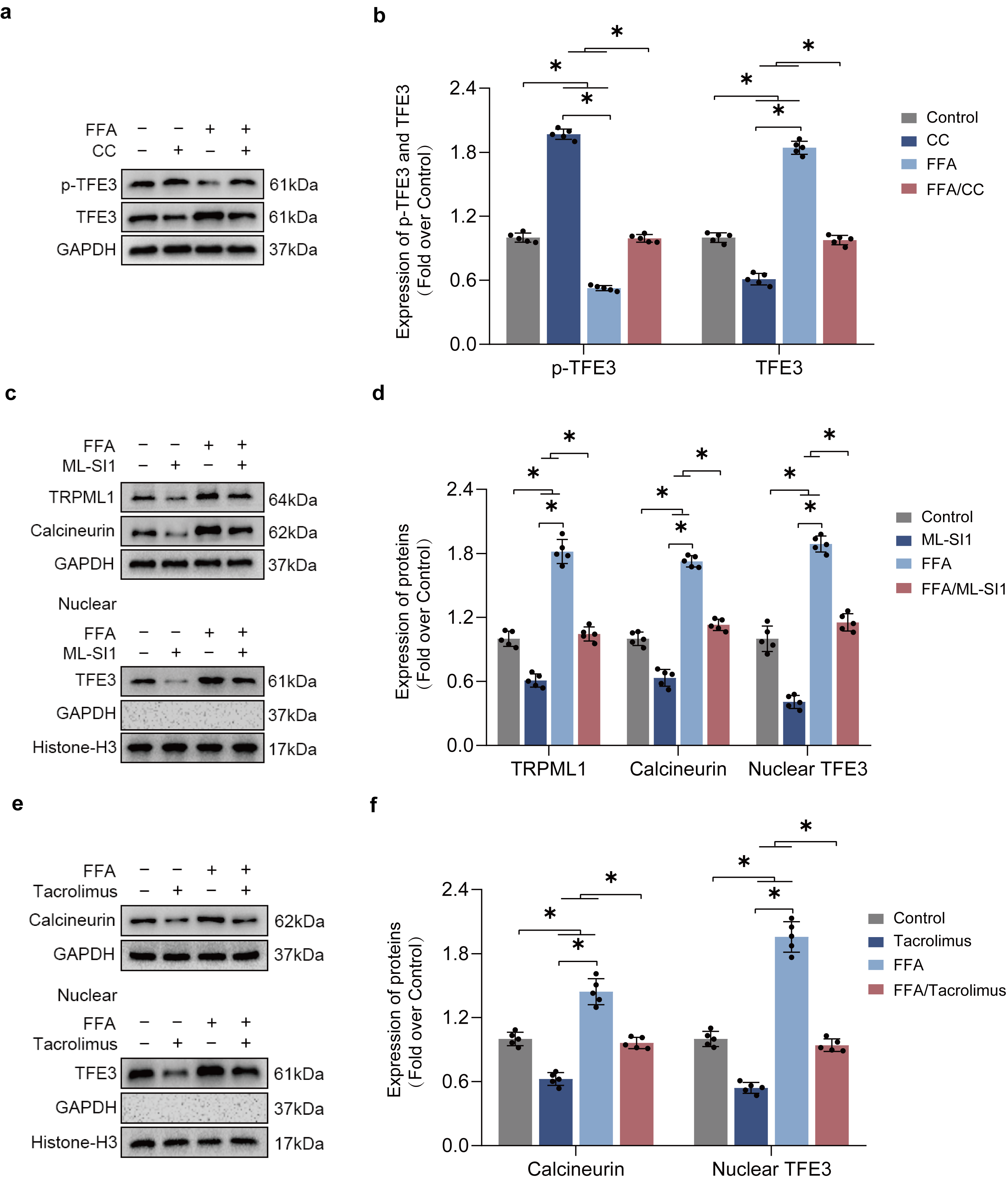


**Figure S6. FFA promotes ischemic flap survival by enhancing TFE3 activity via the AMPK-TRPML1-Calcineurin pathway**

**(a–b)** WB analysis of TRPML1, Calcineurin, and TFE3 in the peri-necrotic area from the Control, ML-SI1, FFA, and FFA/ML-SI1 groups. Histone-H3 or GAPDH served as the loading control. Density quantification is shown on the right. **(c–d)** Western blot analysis of Calcineurin and TFE3 in the peri-necrotic area from the Control, Tacrolimus, FFA, and FFA/Tacrolimus groups. Histone-H3 or GAPDH served as the loading control. Density quantification is shown on the right. **(e-f)** Western blot analysis of p-TFE3 in the peri-necrotic area from the Control, CC, FFA, and FFA/CC groups. Histone-H3 or GAPDH served as the loading control. Statistical analysis was performed using ANOVA with least significant difference post hoc tests or Dunnett’s T3 test. The data are presented as the means ± SD; *p < 0.05 denoting substantially different; ns = not significant. FFA: Flufenamic acid, TFE3: Transcription factor E3, WB: western blotting, TRPML1:Transient receptor potential mucolipin 1, CC: Compound C.

**
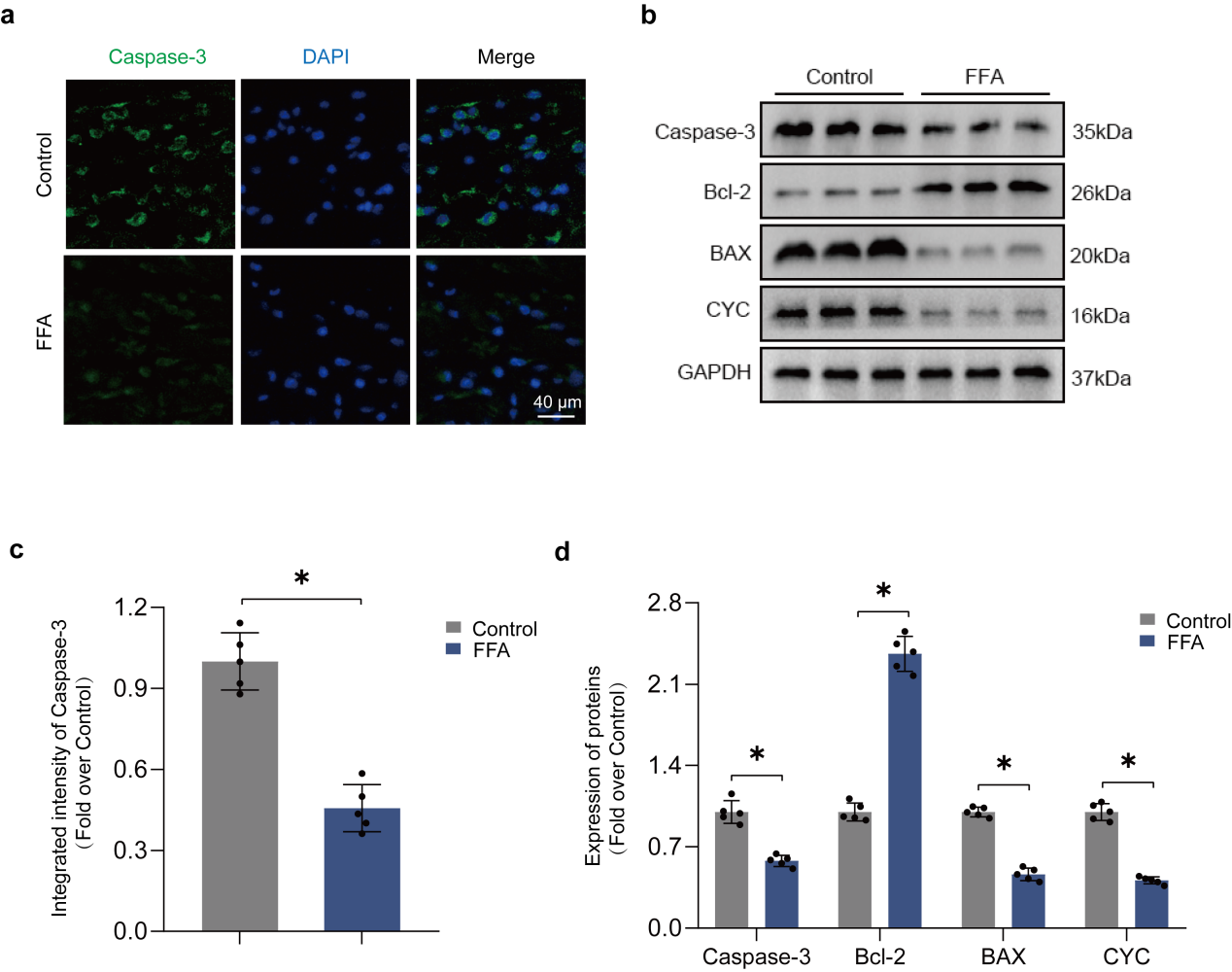
**

**Figure S7. FFA inhibits apoptosis in ischemic flaps.**

1. Skin segments were stained for IF with anti-Caspase-3 (green). Nuclei were stained with DAPI. **(c)** The graph below shows the quantification of anti-Caspase-3 (green) IF. **(b and d)** The peri-necrotic region of ischemic flaps was analyzed for the expression levels of apoptosis-associated proteins using WB. GAPDH was used as the loading control. Two-tailed, unpaired t tests were conducted and the data are presented as the means ± SD; *p < 0.05 denoting substantially different; ns = not significant. FFA: Flufenamic acid, IF: immunofluorescence, WB: western blotting.

**
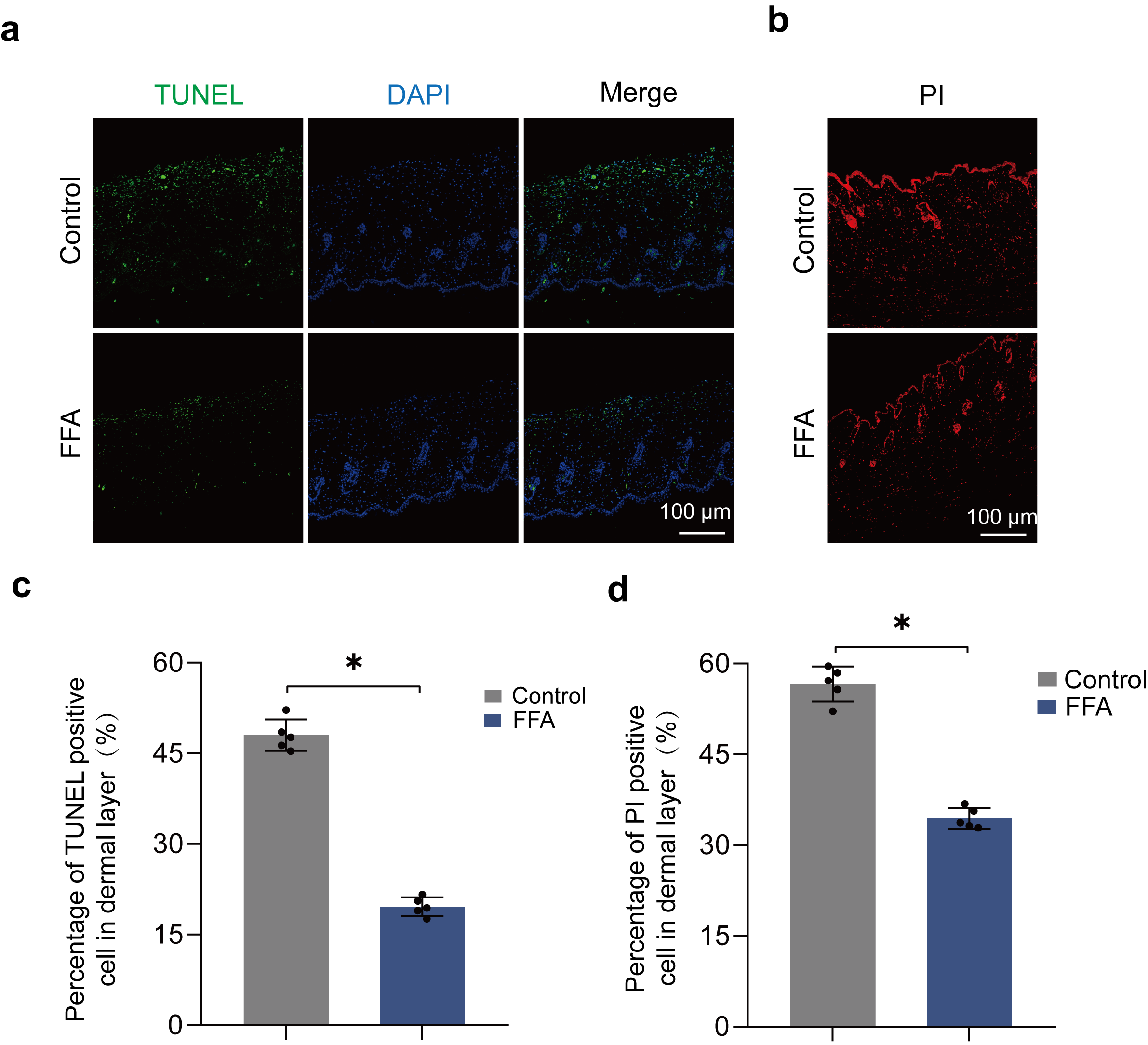
**

**Figure S8. FFA inhibits necrosis in ischemic flaps.**

**(a–b)** Representative images of TUNEL and PI assays detect cell death in the peri-necrotic area of skin flaps from the Control and FFA groups on the 7th postoperative day. **(c-d)** The graph below displays the quantification of TUNEL (green)-positive cells and PI (red)-positive cells. Two-tailed, unpaired t tests were conducted and the data are presented as the means ± SD; *p < 0.05 denoting substantially different; ns = not significant. FFA: Flufenamic acid, TUNEL: Terminal deoxynucleotidyl transferase dUTP Nick End Labeling, PI: Propidium iodide.
